# DNA methylation and its effects on gene expression during primary to secondary growth in poplar stems

**DOI:** 10.1186/s12864-020-06902-6

**Published:** 2020-07-20

**Authors:** Yang Zhang, Cong Liu, He Cheng, Shuanghui Tian, Yingying Liu, Shuang Wang, Huaxin Zhang, Muhammad Saqib, Hairong Wei, Zhigang Wei

**Affiliations:** 1 State Key Laboratory of Tree Genetics and Breeding, Northeast Forestry University, Harbin, Heilongjiang 150040 People’s Republic of China; 2 Research Center of Saline and Alkali Land of State Forestry and Grassland Administration, Chinese Academy of Forestry, Beijing, 100091 People’s Republic of China; 3 Institute of Soil and Environmental Sciences, University of Agriculture, Faisalabad, 38000 Pakistan; 4 College of Forest Resources and Environmental Science, Michigan Technological University, Houghton, MI 49931 USA

**Keywords:** DNA methylation, Gene expression, Primary stems, Transition stems, Secondary stems, *Populus trichocarpa*

## Abstract

**Background:**

As an important epigenetic mark, 5-methylcytosine (5mC) methylation is involved in many DNA-dependent biological processes and plays a role during development and differentiation of multicellular organisms. However, there is still a lack of knowledge about the dynamic aspects and the roles of global 5mC methylation in wood formation in tree trunks. In this study, we not only scrutinized single-base resolution methylomes of primary stems (PS), transitional stems (TS), and secondary stems (SS) of *Populus trichocarpa* using a high-throughput bisulfite sequencing technique, but also analyzed the effects of 5mC methylation on the expression of genes involved in wood formation.

**Results:**

The overall average percentages of CG, CHG, and CHH methylation in poplar stems were ~ 53.6%, ~ 37.7%, and ~ 8.5%, respectively, and the differences of 5mC in genome-wide CG/CHG/CHH contexts among PS, TS, and SS were statistically significant (*p* < 0.05). The evident differences in CG, CHG, and CHH methylation contexts among 2 kb proximal promoters, gene bodies, and 2 kb downstream regions were observed among PS, TS, and SS. Further analysis revealed a perceptible global correlation between 5mC methylation levels of gene bodies and transcript levels but failed to reveal a correlation between 5mC methylation levels of proximal promoter regions and transcript levels. We identified 653 and 858 DMGs and 4978 and 4780 DEGs in PS vs TS and TS vs SS comparisons, respectively. Only 113 genes of 653 DMGs and 4978 DEGs, and 114 genes of 858 DMGs and 4780 DEG were common. Counterparts of some of these common genes in other species, including *Arabidopsis thaliana*, are known to be involved in secondary cell wall biosynthesis and hormone signaling. This indicates that methylation may directly modulate wood formation genes and indirectly attune hormone signaling genes, which in turn impact wood formation.

**Conclusions:**

DNA methylation only marginally affects pathway genes or regulators involved in wood formation, suggesting that further studies of wood formation should lean towards the indirect effects of methylation. The information and data we provide here will be instrumental for understanding the roles of methylation in wood formation in tree species.

## Background

Wood is the most abundant biomass produced by plants, especially trees, and can serve as a renewable resource for energy, pulp, paper products, and building materials [1]. In most trees, wood originates from vascular cambium, the secondary meristem located between tree barks and woody trunks; vascular cambium produces undifferentiated xylem mother cells inwardly and bark cells outwardly. For this reason, cambium activity is the most important determining factor for wood accumulation. Present knowledge indicates that the differentiation of vascular cambium into xylem mother cells is controlled by plant hormones and HD-ZIP III transcription factors [2]. After that, xylem mother cells undergo a series of biological processes, including cell division and expansion, secondary wall formation, lignification, and finally programmed cell death, to produce secondary xylem, known as wood [3, 4]. The coordinated activation of secondary wall biosynthesis in xylem mother cells to produce wood is mediated by a transcriptional network composed of secondary wall NAC and MYB master switches and their downstream transcription factors [1, 2, 5–8]. However, the contribution of epigenetic regulation during this process is still unclear.

DNA methylation, a key epigenetic modification, typically involves the addition of a methyl group to the fifth carbon of cytosine to produce 5-methylcytosine (5mC) in eukaryotic genomes [9, 10]. Although the relationship between DNA methylation and its effect on gene expression is complex [11, 12], an increasing body of evidence suggests that DNA methylation plays a role in various biological processes during plant growth and development [12, 13], such as morphogenesis [14], gender determination [15, 16], vegetative propagation [17], and response to abiotic stress [18–21]. In plants, cytosine methylation is primarily found in three sequence contexts: CG, CHG, and CHH (where H = A, T or C) [22]. Moreover, DNA methylation exhibits tissue specific patterns in plants. For example, in *Arabidopsis thaliana*, about 6% of cytosines are methylated in immature floral tissues [23], while 24% CG, 6.7% CHG, and 1.7% CHH are methylated in young plants [24]. In rice, whole genome methylation patterns are similar among mature leaves, embryos, and seedling shoots and roots, but hypomethylation levels are correlated with expression levels of genes that are preferentially expressed in endosperm [25]. Patterns of 5mC in long terminal repeat (LTR) transposable elements differ between rice leaves and roots [26] and affect neighboring gene expression in *A. thaliana* [27, 28]. Tissue-specific characteristics of genome methylation are also evident in natural populations of Chinese white poplar [29]. Although DNA methylation is purported to play an important role in wood formation [30, 31], the mechanisms by which DNA methylation alter the expression of xylogenetic genes have not been elucidated. Moreover, tissue-specific methylation patterns in the transitional zones between vascular cambium and secondary wood have not been characterized.

The transition from primary to secondary growth can be easily observed in the stems of less than one-year old poplar trees with multiple developmental stages. For instance, stems near apical meristems are generally soft and green due to the presence of a multitude of cells with primary cell walls; in contrast, stems in basal portions are stiff and woody owing to the presence of a large fraction of secondary xylem cells that have undergone cell wall thickening and lignification. Stems in the middle are in a transitional stage between primary and secondary growth. For this reason, vertical segments of developing stems from less than one-year old trees constitute an ideal experimental system for investigating epigenetic regulatory mechanisms of wood formation [32, 33]. To date, no focused study of DNA methylation and its effects on gene expression in different developmental stages of stems has been conducted in tree species. In this study, we generated high coverage genome-wide maps of cytosine methylation at single-nucleotide resolution and transcriptomic profiles of *Populus trichocarpa* stems in various developmental stages varying from predominantly primary to secondary growth. This study was designed to collect data and gain insight into four problems: (i) the genomic landscape of the different developmental stem methylomes; (ii) the changes in the methylomes associated with different stem developmental stages; (iii) an evaluation of relationships between methylome changes and expression of wood formation genes; (iv) the identification of wood formation genes that are subjected to epigenetic regulation. The epigenetic and RNA-seq data acquired constitute valuable genetic resources, and the results and conclusions drawn from the data and analysis will be instrumental for further studies of both the epigenetic and molecular regulatory mechanisms of wood formation.

## Results

### Morphological and histochemical changes in *P. trichocarpa* stems

**Fig. 1:**
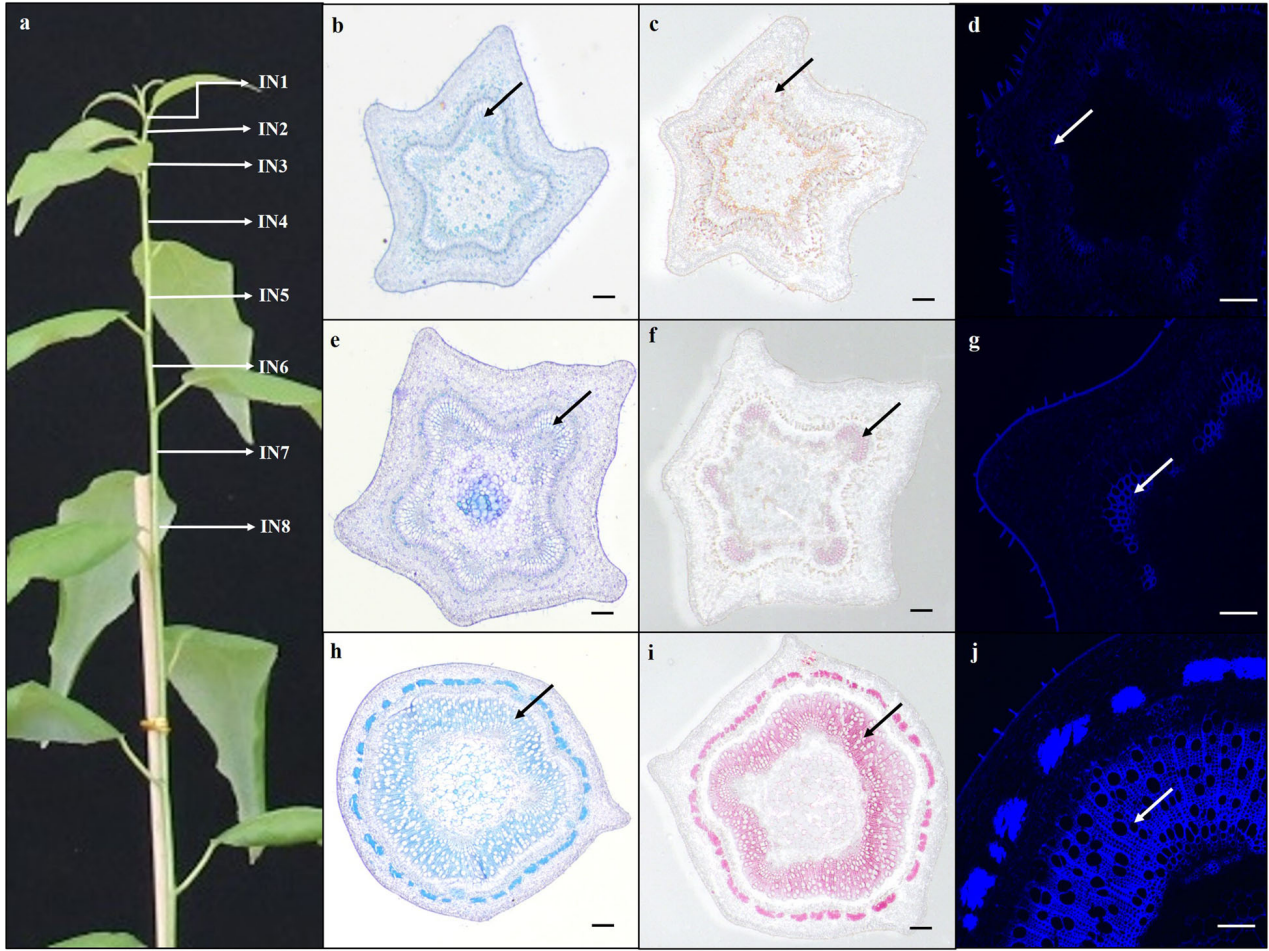
Anatomical and histochemical analyses in *Populus trichocarpa* stems of different developmental stages. **a** An illustration of stem segments in a 90-day-old *Populus trichocarpa* sample plant used as study material. The number of each internode (IN) is indicated from the apical bud to the base of the stem. **b**, **e**, and **h** represent toluidine blue O-stained transverse sections from the internodes two (IN2), four (IN4), and eight (IN8), respectively. **c**, **f**, and **i** are phloroglucinol-HCl-stained transverse sections from IN2, IN4, and IN8, respectively. **d**, **g**, and **j** represent calcofluor white-stained transverse sections from IN2, IN4, and IN8 under UV light, respectively. The arrowheads represent changes in xylem of *P. trichocarpa* stems. Scale bars = 200 μm

To verify the rationality of the classification of the main stems of poplar into different developmental stages using the plastochron indices method, we determined the developmental stages of internodes two (IN2), four (IN4), and eight (IN8) using histochemical staining. Toluidine blue-O and phloroglucinol-HCl were used to stain lignin while calcofluor white was used to stain cellulose in xylem vessel elements. Because the vascular bundles in IN2 comprised mainly of primary xylem and phloem tissues that were formed from procambial cells, toluidine blue-O and phloroglucinol-HCl staining in the cross sections of IN2 were nearly undetectable (Fig. 1b and c), and calcofluor white staining in IN2 sections was also weak (Fig. 1d). In IN4, the secondary vascular cambium has emerged and produced secondary walls. As a result, the lignin stained by either toluidine blue-O or phloroglucinol-HCl was clearly discernible (Fig. 1e and f), and the cellulose stained by calcofluor white was also more obvious (Fig. 1g) than in IN2 (Fig. 1d). In the stem segments of IN8, the secondary xylem had increasingly accumulated, phloem fibers had emerged, and both were lignified. As a result, the intensities of toluidine blue-O, phloroglucinol-HCl, and calcofluor white staining in the cross sections of IN8 (Fig. 1h-j) were much more striking than in IN2 or IN4 (Fig. 1b-g). Therefore, IN2, IN4, and IN8, representing the stages of primary stems (PS), transitional stems (TS), and secondary stems (SS) from primary growth to secondary growth, respectively, were used for further analysis. To avoid getting into a state of uncertainty by virtue of using multiple cross-section tissues, we harvested only the primary xylem upon peeling tree bark and focused our studies on DNA methylation and genomic aspects of xylogenesis.

### The expression levels of genes involved in DNA methylation and demethylation in *P. trichocarpa* stems

**Fig. 2:**
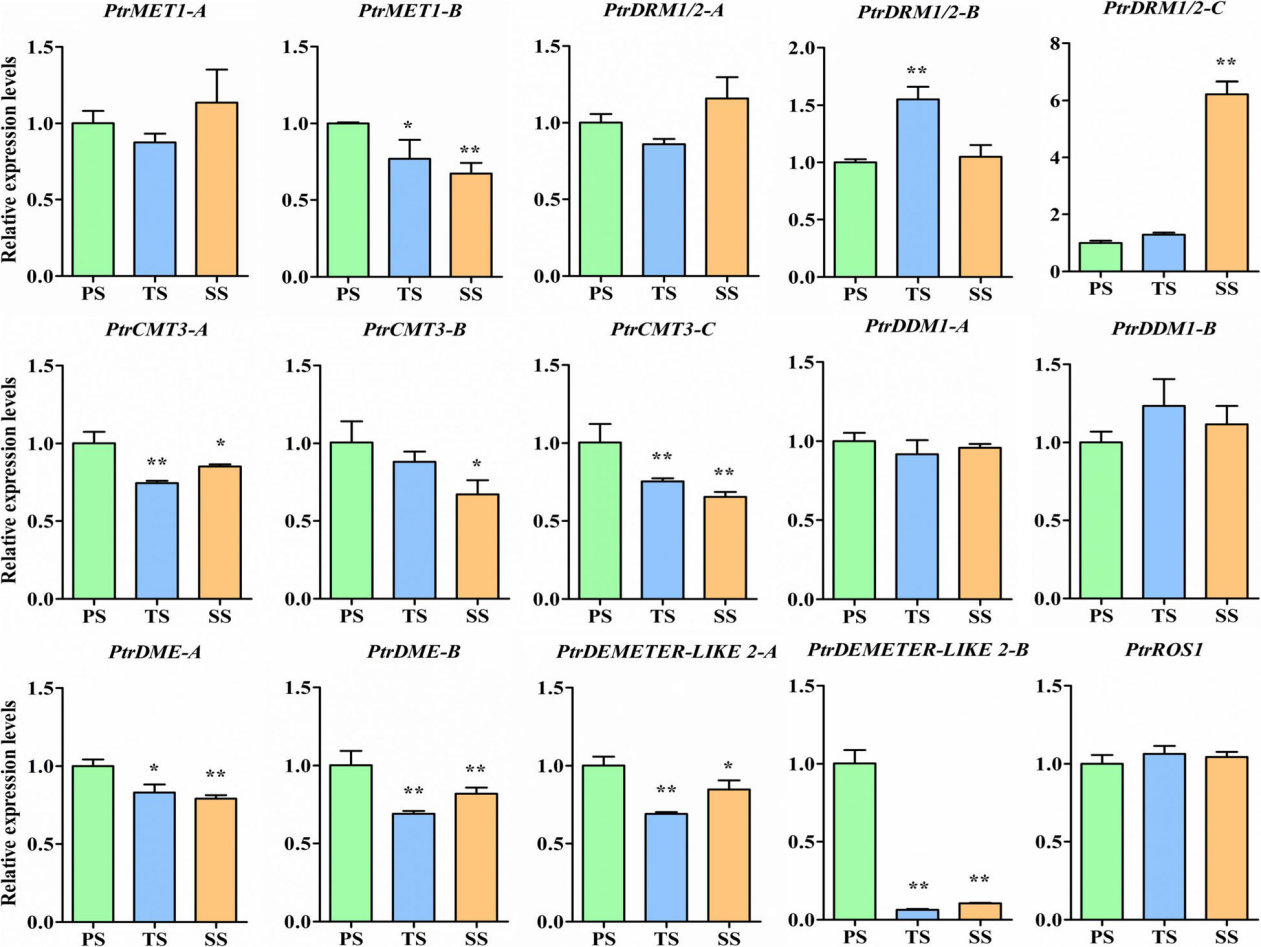
qRT-PCR analysis of genes encoding DNA methyltransferases and demethylases in stems of *Populus trichocarpa*. Transcript level of the actin gene *in P. trichocarpa* was used as an endogenous control to normalize expression values of other genes in primary stems (PS), transitional stems (TS), and secondary stems (SS). Bars and standard errors represent the means and standard errors, respectively, of three biological replicates. Each biological replicate was represented by an independent RNA extraction in two technical replicates. The data were analyzed using one-way ANOVA using SPSS 21. Significant differences among different comparisons were determined with Duncan’s multiple range test and significant and highly significant differences are indicated by *(*P* < 0.05) and **(*P* < 0.01), respectively

To determine whether variations in DNA methylation exist among PS, TS, and SS, we first used qRT-PCR to globally scrutinize the expression levels of genes involved in DNA methylation. We focused on the DNA methylation genes *PtrMET1A/B*, *PtrDRM1/2-A-C, PtrCMT3-A*-C, and *PtrDDM1-A*/*B* and the DNA demethylation genes *PtrDME-A/B, PtrDEMETER-LIKE 2-A/B*, and *PtrROS1.* As shown in Fig. 2*,* the expression levels of *PtrMET1-B*, *PtrCMT3-A*, and *PtrCMT3-C* were significantly different among PS, TS, and SS. *PtrDRM1/2-C* had significantly higher and *PtrCMT3-B* had significantly lower expression levels in SS than in both PS and TS. The expression level of *PtrDRM1/2-*B in TS was significantly higher than in PS and SS. However, there were no statistically significant differences in the expression levels of *PtrMET1-A, PtrDRM1/2-A, PtrDDM1-A*, and *PtrDDM1-B* among PS, TS, and SS. Of the expressed genes involved in DNA demethylation, only *PtrDME-A* and *PtrDEMETER-LIKE 2-A* exhibited significant differences in expression levels among PS, TS, and SS*.* The expression levels of *PtrDME-B* and *PtrDEMETER-LIKE 2-B* in TS and SS exhibited significant differences compared to PS. However, the expression levels of these three demethylation genes had no obvious differences between TS and SS. Moreover, there were no significant differences in the expression levels of *PtrROS1* among PS, TS, and SS. In summary, the differential expression of these genes across three developmental stages suggests that genomic DNA methylation patterns may be altered during the wood formation process.

### Whole-Genome Bisulfite Sequencing (WGBS) of the *P. trichocarpa* genome

Variations in the expression levels of genes involved in DNA methylation suggest that genomic DNA methylation levels might be different across PS, TS, and SS. To investigate the genomics methylation levels of poplar in the stems of different developmental stages, we performed bisulfite sequencing of genomic DNA extracted from PS, TS, and SS using the Illumina HiSeq 2500 platform. We then decoded and analyzed the corresponding methylomes. A total of 99.5–115.6 million raw sequencing paired reads were obtained for each biological replicate (Table 1), covering the whole genome of *P. trichocarpa* with a depth between 30.35–34.29-fold. Raw reads were then subjected to a series of filtering criteria to ensure data quality, and 99.82–99.88% of the reads were retained for further analysis. The reads from each sample were mapped to the *P. trichocarpa* reference genome with a mapping rate of 75.74–80.27% (Table 1).

**Table 1:** Description of the bisulfite sequencing (BS-Seq) data of early developing stems (3-month-old) in *Populus trichocarpa*

Sample		Raw reads	Clean reads	Sequence Depth	Mapped Reads	Mapped Rate (%)
**PS**	Rep1	105,344,898	105,190,862 (99.85%)	30.79	80,968,657	76.97
	Rep2	99,553,450	99,425,308 (99.87%)	30.35	79,809,380	80.27
	Rep3	107,558,146	107,425,046 (99.88%)	32.16	84,583,644	78.74
**TS**	Rep1	107,666,760	10,749,4930 (99.84%)	31.51	82,870,707	77.09
	Rep2	109,561,376	109,409,538 (99.86%)	31.51	82,864,569	75.74
	Rep3	115,622,622	115,474,758 (99.87%)	34.14	89,790,043	77.76
**SS**	Rep1	113,318,946	113,174,330 (99.87%)	34.29	90,186,749	79.69
	Rep2	107,441,448	107,246,874 (99.82%)	32.09	84,393,945	78.69
	Rep3	115,564,860	115,357,460 (99.82%)	33.95	89,294,118	77.41

Note: primary stems (PS), transitional stems (TS), and secondary stems (SS)

### DNA methylation landscapes of *P. trichocarpa* genome

**Fig. 3:**
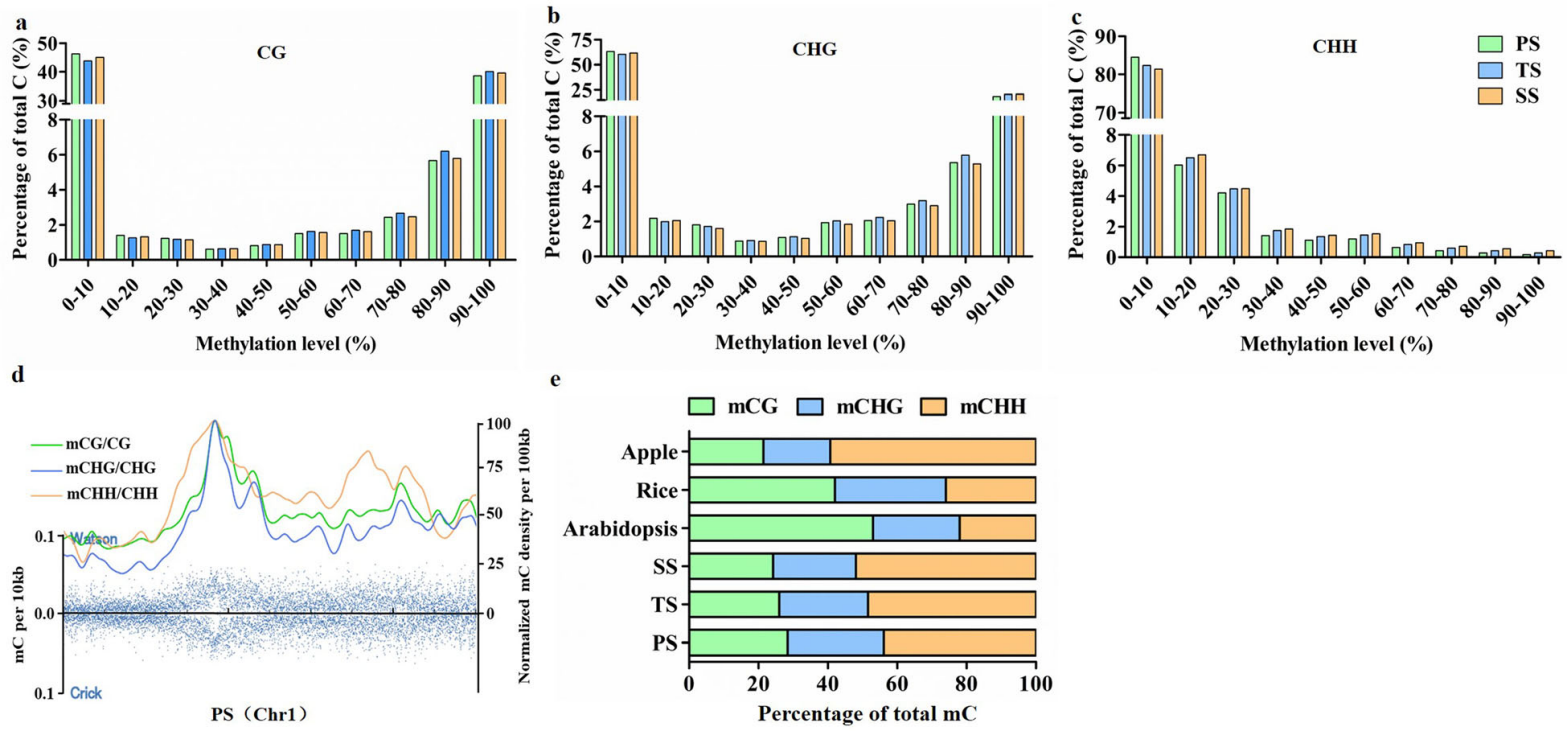
The *Populus trichocarpa* epigenome. The percentage of methylated cytosine (mC) distribution in each sequence context. **a** CG methylation; **b** CHG methylation; **c** CHH methylation in primary stems (PS), transitional stems (TS), and secondary stems (SS). The y-axis indicates the percentage of methylated cytosines according to each methylation level range, which is shown on the x-axis. **d** Distribution of 5-methylcytosine density on chromosome 1 in PS. **e** Relative proportions of mCs in three sequence contexts (CG, CHG, and CHH) in *P. trichocarpa* (PS, TS, and SS), *Arabidopsis thaliana*, rice, and apple

Conversion rates were calculated by aligning reads to the unmethylated lambda DNA added to the total DNA before applying bisulfite treatment. Conversion rates of genomic DNA of PS, TS, and SS were on average 99.51, 99.52, and 99.50%, respectively, these rates were used to conduct binomial tests to exclude those 5mCs that may be the result of non-conversion of cytosines in our bisulfite treatment or sequencing errors resulting from the base calling process. Then, we obtained on average 14,773,999, 16,392,099, and 16,852,157 mCs for the PS, TS, and SS genomes, respectively (Additional file 1). The PS genome harbored ~ 11.96, 47.64, 28.85, and 4.85% methylated C at the total sequenced C, CG, CHG, and CHH sites, respectively. Likewise, the TS genome contained ~ 13.33, 49.89, 31.53, and 5.88% methylated C while the SS genome contained ~ 13.55, 48.80, 30.80, and 6.46% methylated C, respectively, at the total sequenced C, CG, CHG, and CHH sites (Additional file 1). We also found that, regardless of developmental stage (PS, TS, or SS), ~ 45% of CG and ~ 65% of CHG sites were lowly methylated (0–10%) while ~ 40% of CG and ~ 24% of CHG sites were highly methylated (90–100%) (Fig. 3a and b); in contrast, ~ 83% of CHH sites were lowly methylated (0–10%) (Fig. 3c) and less than 1% of CHH sites were highly methylated (90–100%). These results suggest that nearly half of CG methylation sites are either hypomethylated or hypermethylated, nearly two thirds of CHG methylation sites are hypermethylated, and the majority of CHH sites are hypomethylated in poplar stems.

As an important methylation characterisitcs of a genome, the proportions of mCG, mCHG, and mCHH on total mC sites have species and tissues specificity. Thus, we not only identified the distribution patterns of mC sites in mCG, mCHG, and mCHH contexts among PS, TS, and SS, but we also compared the mC site distribution patterns of poplar stems with *A. thaliana* [23, 24, 34], rice [35], and apple [36]. The overall distribution patterns of mC sites in mCG, mCHG, and mCHH were illustrated using Chromosome 1 in the PS genome (Fig. 3d). The distribution of mCs in the other 18 chromosomes were also determined on sense and antisense strands (Additional files 2, 3, and 4).

We found that PS, TS, and SS exhibited nearly same distribution patterns of total mC sites in three methylation contexts as those in apple on the whole. However, the mCs exhibited different distribution patterns in *P. trichocarpa* compared to *A. thaliana* and rice, especially in mCG and mCHH contexts (Fig. 3e). In PS, 5mC was found more frequently at CHH sites (43.87%) than at CG (28.42%) or CHG (27.71%) sites. In TS and SS, CHH methylation rates increased to 48.47 and 51.93%, respectively, indicating that the CHH methylation rate increases in accordance with the progression of secondary growth and development. Accordingly, the CG methylation rates in TS and SS decreased to 25.95 and 24.22%, respectively, while the CHG methylation rates decreased to 25.61 and 23.87%, respectively (Additional file 5), suggesting that the levels of these two methylation contexts negatively correlate with the progression of secondary growth. The comparisons of PS, TS, and SS among CG, CHG, and CHH methylation rates revealed that there were significant differences in mCG, mCHG, and mCHH contexts among PS, TS, and SS (Additional file 6).

**Fig. 4:**
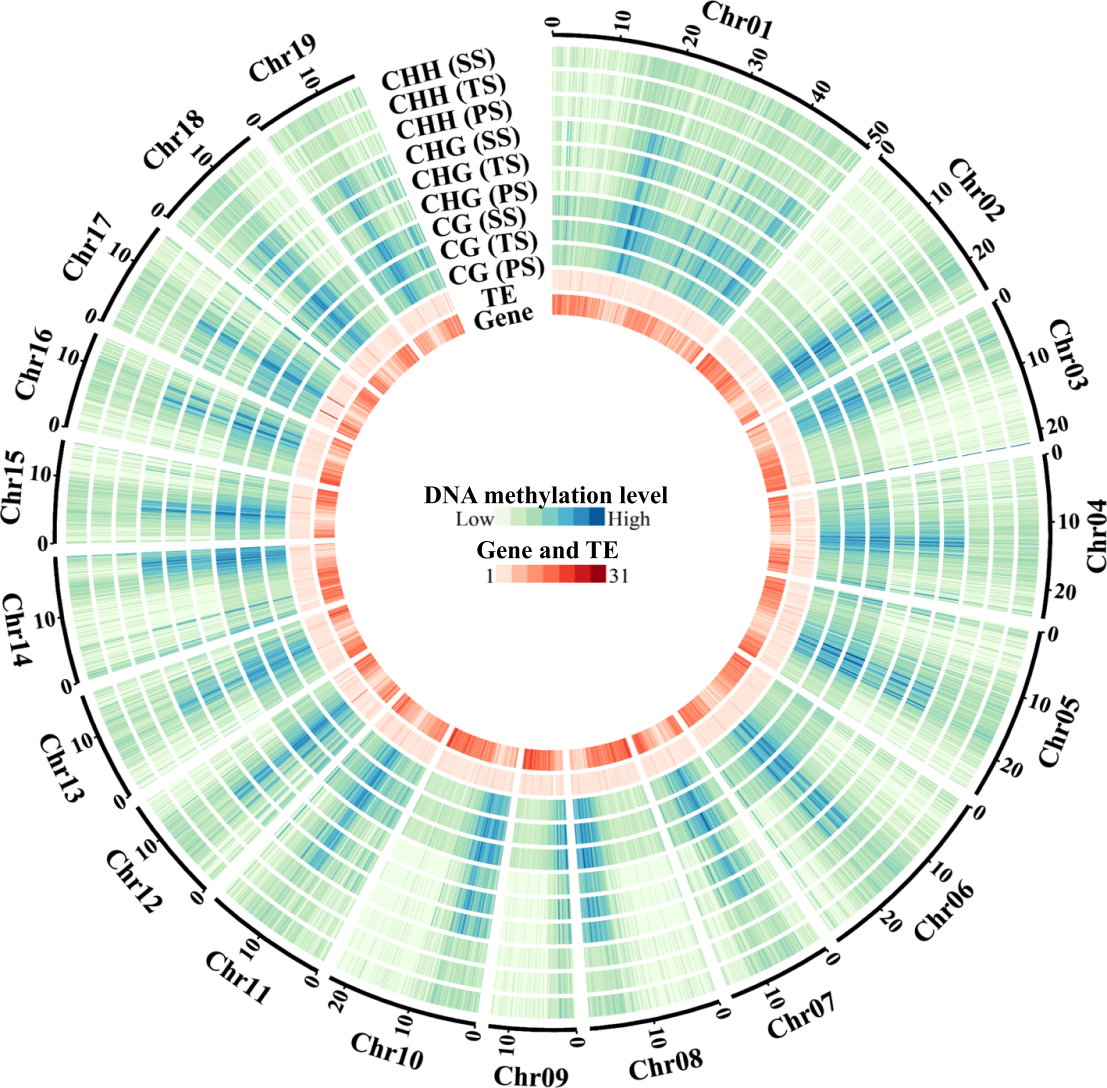
Circos plots of methylation patterns in the *Populus trichocarpa* genome. Tracks shown in an outward order are: Track 1 (innermost), gene; Track 2, transposable element (TE); Tracks 3–5, density plots of 5-methylcytosine (5mC) in CG contexts in primary stems (PS), transitional stems (TS), and secondary stems (SS), respectively; Tracks 6–8, density plots of 5mC in CHG contexts in PS, TS, and SS, respectively; Tracks 9–11, density plots of 5mC in CHH contexts in PS, TS, and SS, respectively

Regardless of PS, TS, and SS, the poplar genome showed a relatively lower methylation level within gene-rich regions compared to a relatively high degree of methylation within transposable element (TE)-rich regions (Fig. 4). Moreover, the gene-rich regions with few or no TEs exhibited a relatively less methylation levels (Fig. 4) as compared to the gene-rich regions with more TEs. We did not find large-scale differences in the genomes of *P. trichocarpa* stems from different stages.

### Genomic methylation patterns in *P. trichocarpa* stems

**Fig. 5:**
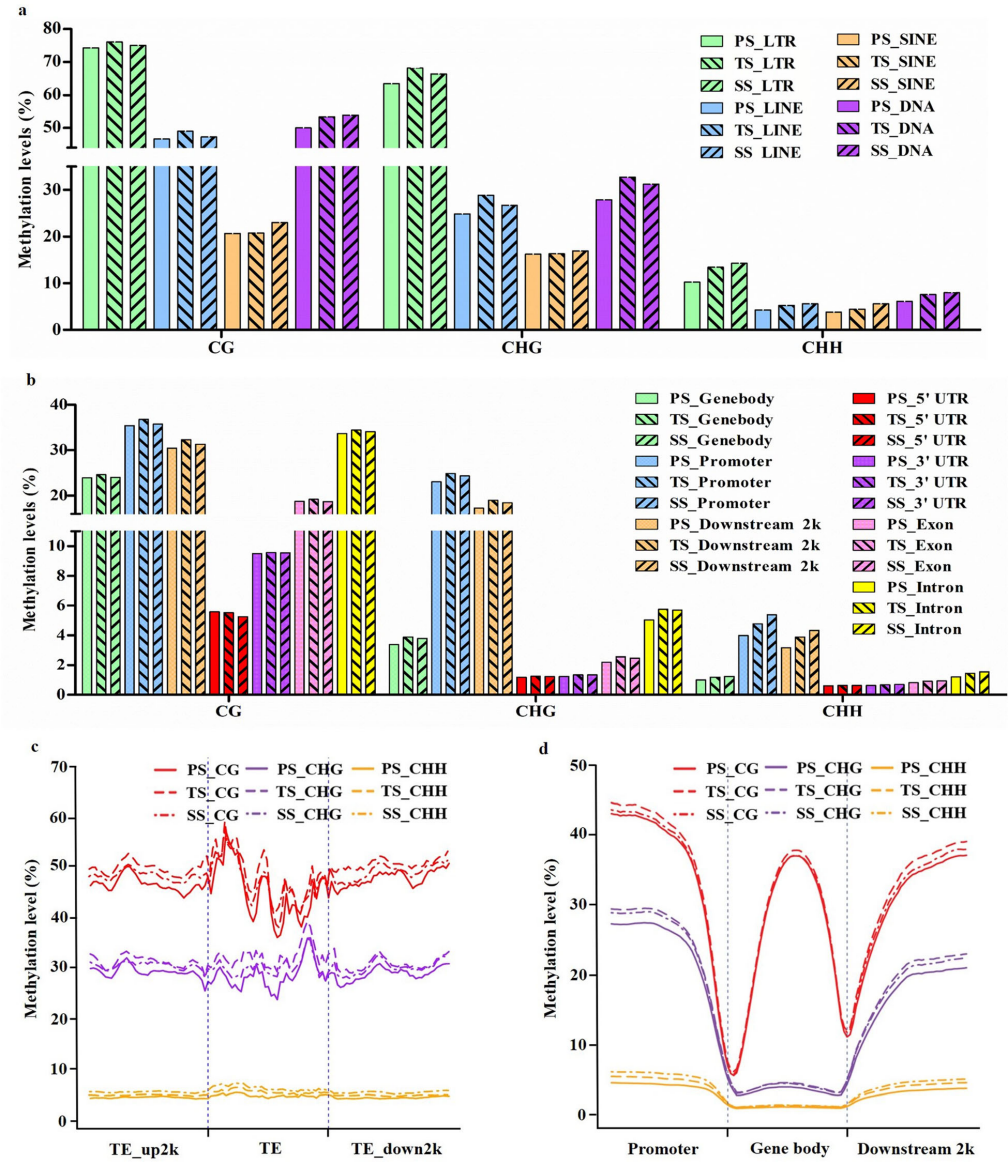
DNA methylation patterns in different genic regions. **a** Methylation levels in different types of transposable elements (TEs), including long terminal repeats (LTR), long interspersed nuclear elements (LINE), short interspersed nuclear elements (SINE), and DNA transposons (DNA) in primary stems (PS), transitional stems (TS), and secondary stems (SS). **b** Methylation levels of gene features, including promoter, 2 kb downstream region, and gene body regions with 5’UTR, exon, intron, and 3’UTR regions in stems of poplar. **c** Methylation levels among TE regions and their 2 kb upstream and downstream regions in stems of poplar. **d** Distribution of methylation levels among gene features, including promoter, gene body, and 2 kb downstream regions in stems of poplar. The y-axis indicates methylation levels

Given the existence of tissue level variation in DNA methylation in the *P. trichocarpa* genome [30], we further explored the methylation profiles of PS, TS, and SS within different genomic regions; this included different genic and intergenic regions, especially repetitive regions containing various transposable elements (TEs) such as long terminal repeats (LTR), long interspersed nuclear elements (LINE), short interspersed nuclear elements (SINE), and DNA transposons (DNA). In PS, TS, and SS, CG and CHG methylation levels were higher than CHH methylation levels in each of the genomic regions mentioned above (Fig. 5a and b). There were significant differences in methylation in the CG and CHG contexts when various specific genomic regions were compared. For example, PS/TS/SS_Gene body of CG verse PS/TS/SS_Gene body of CHG methylation (Fig. 5b). In addition, methylation levels in CG, CHG, and CHH contexts were slightly higher in TS and SS than in PS (Fig. 5a and b). LTRs had the highest methylation levels in all three methylation contexts (CG, CHG, and CHH) in PS, TS, and SS (Fig. 5a). In contrast, SINEs had the lowest methylation levels in all three contexts and stages of stem development. LINEs had modest methylation in all three contexts of methylation and also three developmental stages. Further research found that LTR Gypsy, LTR Caulimovirus, LINE L1, and DNA CMC-EnSpm, the predominant type of transposable element sequence in *P. trichocarpa* genomes [37], had higher methylation levels than others in the stems of *P. trichocarpa* (Additional file 7). In addition, the LTR Copia and LTR Gypsy super families had no distinct differences in their methylation levels across PS, SS, and TS, which resembles their relatively invariant methylation levels across seven tissues (vegetative bud, male inflorescence, female catkin, leaf, root, xylem, and phloem) of *P. trichocarpa* as observed earlier [30]. Among different genic regions, the 5’UTR and 3’UTR had much lower methylation levels than other regions; promoters and 2 kb downstream regions had higher methylation levels than other regions in all three methylation contexts in PS, TS, and SS (Fig. 5b).

We also found that methylation levels changed during stem development in *P. trichocarpa.* In TEs and genic regions, methylation levels of CG, CHG, and CHH contexts were increased in TS and SS compared to PS (Fig. 5c and d), and methylation levels in CG and CHG contexts were highest in TS. However, the methylation levels in CHH contexts were highest in SS. As for TE regions, the CG context had the highest methylation level, and CHH had the lowest level of the three methylation contexts in PS, TS, and SS (Fig. 5c). In both CG and CHG contexts, TEs had higher methylation levels than 2 kb upstream and 2 kb downstream regions. However, there were no conspicuous differences in CHH methylation levels among TEs, 2 kb upstream, and 2 kb downstream regions in all three tissues. Additional studies showed that several TE super families, including LINE L1, DNA CMC-EnSpm, DNA hAT-Tag1, and DNA hAT-Tlp100, had higher CG and CHG methylation levels in TEs than their 2 kb upstream and 2 kb downstream regions (Additional file 8). In addition, the LTR Copia and LTR Gypsy super families had no distinct differences in three CG, CHG, and CHH methylation levels among TEs and their 2 kb upstream and downstream regions in all three tissues.

As shown in Fig. 5d, the three methylation contexts were ranked consistently from highest to lowest methylation as CG, CHG, and CHH, no matter which genic regions or tissue types were considered. Moreover, promoter regions had higher methylation levels compared with either gene bodies or 2 kb downstream regions in all three stem tissues. It was notable that gene bodies had lower methylation levels, especially for CHG and CHH, as compared with either the promoter regions or the 2 kb downstream regions. To compare methylation levels in the three genomic contexts in different genic regions across multiple tissues, multiple comparison testing was conducted; significant differences among difference comparisons are provided in Additional file 9. Within the promoter regions, there were no significant differences in CG methylation levels among PS, TS, and SS (Additional file 9A). There were significant differences in CHG methylation levels between PS and TS and also between PS and SS (Additional file 9B) and significant differences in CHH methylation levels among PS, TS, and SS (Additional file 9C). Within the gene bodies, there were significant differences in CG contexts between TS and SS (Additional file 9D), in CHG contexts between PS and TS and between PS and SS (Additional file 9E), and in CHH contexts among PS, TS, and SS (Additional file 9F). Within the 2 kb downstream regions, there were significant differences in CG methylation levels between PS and SS, in CHG between PS and TS and between PS and SS, and in CHH methylation levels between any combination of PS, TS, and SS (Additional file 9G-I). However, despite being statistically significant, the variations observed in methylation levels are limited.

### Relation between DNA methylation and gene expression

**Fig. 6:**
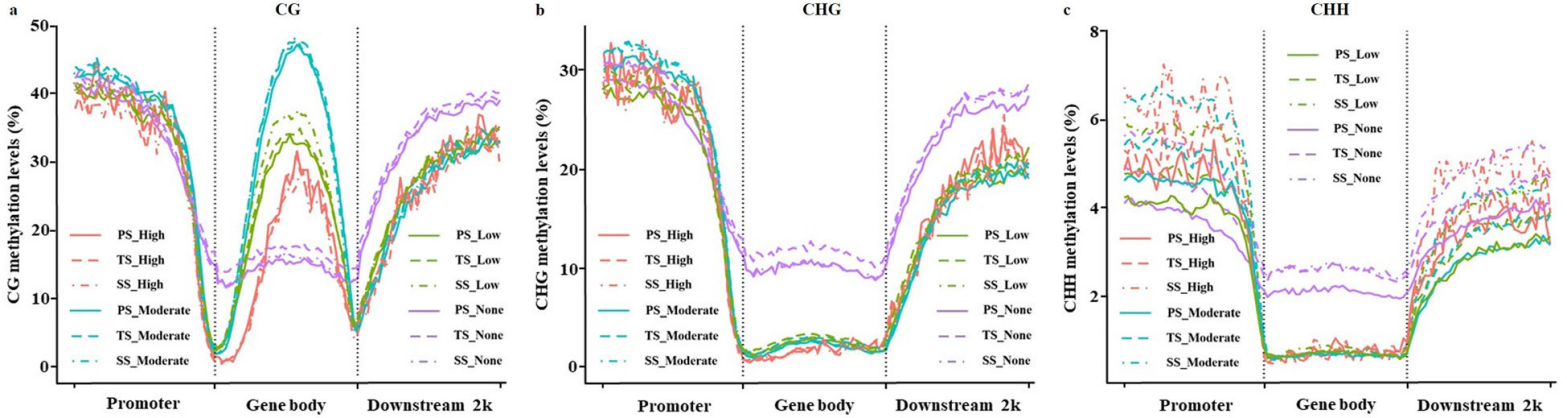
Relationship between gene expression and methylation (5-methylcytosine) in *Populus trichocarpa* stems at different developmental stages. **a**, **b**, and **c** Distributions of CG, CHG, and CHH methylation levels, respectively, in various genic regions including 2 kb promoters, gene bodies, and 2 kb downstream regions in primary stems (PS), transitional stems (TS), and secondary stems (SS). The y-axis indicates methylation levels. Methylation levels were partitioned based on gene expression levels. None represents non-expressed genes (FPKM ≤1); Low represents minimally expressed genes (1 < FPKM ≤10); Moderate represents moderately expressed genes (10 < FPKM ≤100); and High represents highly expressed genes (FPKM ≥100)

To investigate the potential influence of gene methylation on gene expression during wood formation, transcriptome profiling of PS, TS, and SS was conducted using the same materials used for methylome analysis. Based on the FPKM gene expression levels, we classified all genes into non-expressed or expressed genes; the latter were further divided into three groups based on their expression level: low, moderate, or high. We then scrutinized the differences in CG, CHG, and CHH methylation levels within the proximal promoters, gene bodies, and 2 kb downstream regions of genes having different expression levels. As shown in Fig. 6, the four differentially expressed groups had different methylation levels in the three methylation contexts among proximal promoters, gene bodies, and 2 kb downstream regions. As expected, non-expressed genes had the highest CHG and CHH methylation levels in gene body regions and the highest CG, CHG, and CHH methylation levels within 2 kb downstream regions in PS, TS, and SS. In contrast, non-expressed genes had the lowest CHH methylation levels and moderate CG and CHG methylation levels in upstream 2 kb promoter regions in PS, TS, and SS. Interestingly, regardless of their expression levels, expressed genes had higher CG, but lower CHG and CHH, methylation levels than non-expressed genes in gene bodies in PS, TS, and SS. Expressed genes with moderate expression levels had the highest CG methylation levels within gene bodies, and the highest CG and CHG methylation levels in promoter regions in PS, TS, and SS. These results suggest that genes with different expression levels correspond to different CG, CHG, and CHH methylation levels in different genic regions. Overall, CG and CHG methylation patterns in expressed genes are similar in promoter regions while CHG and CHH methylation patterns in expressed genes are similar in gene bodies and downstream regions.

To further evaluate the relationships between genic methylation and gene expression, genes with transcriptomic profiles were classified into unmethylated (None) and methylated groups; genes in the methylated group were further divided into three subgroups: the bottom third were referred to as the low-methylation subgroup (Low), the middle third as the moderate-methylation subgroup (Moderate), and the top third as the high-methylation group (High) based on their methylation levels. The results suggest that methylations in gene bodies have more obvious correlation on gene expression than those in promoters and 2 kb downstream regions in all three methylation contexts (Additional file 10). The proportion of genes with the lowest methylation levels within promoters and with the highest methylation levels within 2 kb downstream regions were the lowest among the three methylation levels, no matter which type of methylation contexts they were. Moreover, the greatest proportion of genes were moderately methylated in gene bodies in all three methylation contexts. These results suggest that methylation level and context have different effects on gene expression depending on their genic location.

To further study the relationship between genic methylation and gene expression, Spearman correlation analysis was performed between methylation levels in the whole gene frame (bodies ±2 kb flanking regions) and gene expression levels. As shown in Additional file 11, the overall correlation rho was low regardless of the methylation types and the developmental stages. However, the rho can reach 0.25 in gene bodies for CG methylation in PS, TS, or SS. Such a rho may indicate that methylation levels in a small fraction of gene bodies have relatively higher correlation with their expression levels.

### Widespread dynamic gene methylation in *P. trichocarpa* stems

**Fig. 7:**
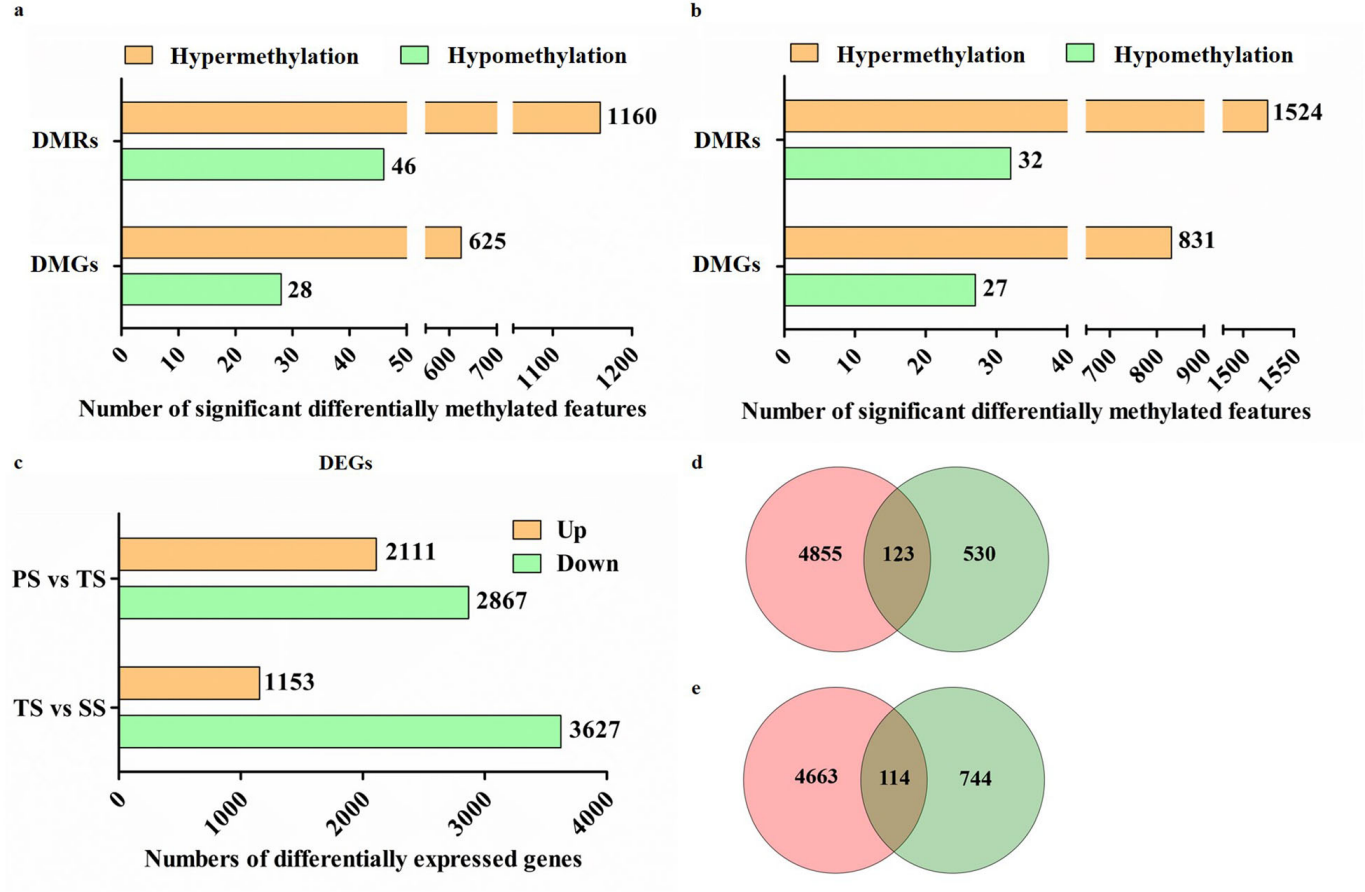
Comparative study of differentially expressed genes (DEGs), differentially methylated regions (DMRs), and differentially methylated genes (DMGs) from primary stems (PS) versus transitional stems (TS) and TS versus secondary stems (SS). **a** The number of DMRs and DMGs in PS vs TS. **b** The number of DMRs and DMGs in TS vs SS. Hypermethylation and hypomethylation refer to increased and decreased methylation levels, respectively. **c** The number of DEGs. Up and down means increased and decreased gene expression, respectively. **d** Venn diagram of DEGs and DMGs from PS vs TS. **e** Venn diagram of DEGs and DMGs from TS vs SS. Pink circles represent DEGs. Green circles represent DMGs. The overlapping shaded areas represent genes that are shared between DEGs and DMGs

To determine the relationship between developmetal stages and methylation, we analyzed the differentially methylated regions (DMRs) between PS and TS and between TS and SS. For PS vs TS, the 1206 DMRs (1160 hypermethylated and 46 hypomethylated regions) (Fig. 7a) overlapped 653 differentially methylated genes (DMGs) (625 hypermethylated and 28 hypomethylated genes). For TS vs SS, the 1556 DMRs (1524 hypermethylated and 32 hypomethylated regions) overlapped the 858 DMGs (831 hypermethylated and 27 hypomethylated genes) (Fig. 7b). At same time, we also identified 4978 differentially expressed genes (DEGs) from PS vs TS and 4780 DEGs from TS vs SS (Fig. 7c). However, there are only 123 common genes between the 653 DMGs and the 4978 DEGs from PS vs TS, and 114 common genes between the 858 DMGs and the 4780 DEGs from TS vs SS (Fig. 7d and e).

**Fig. 8:**
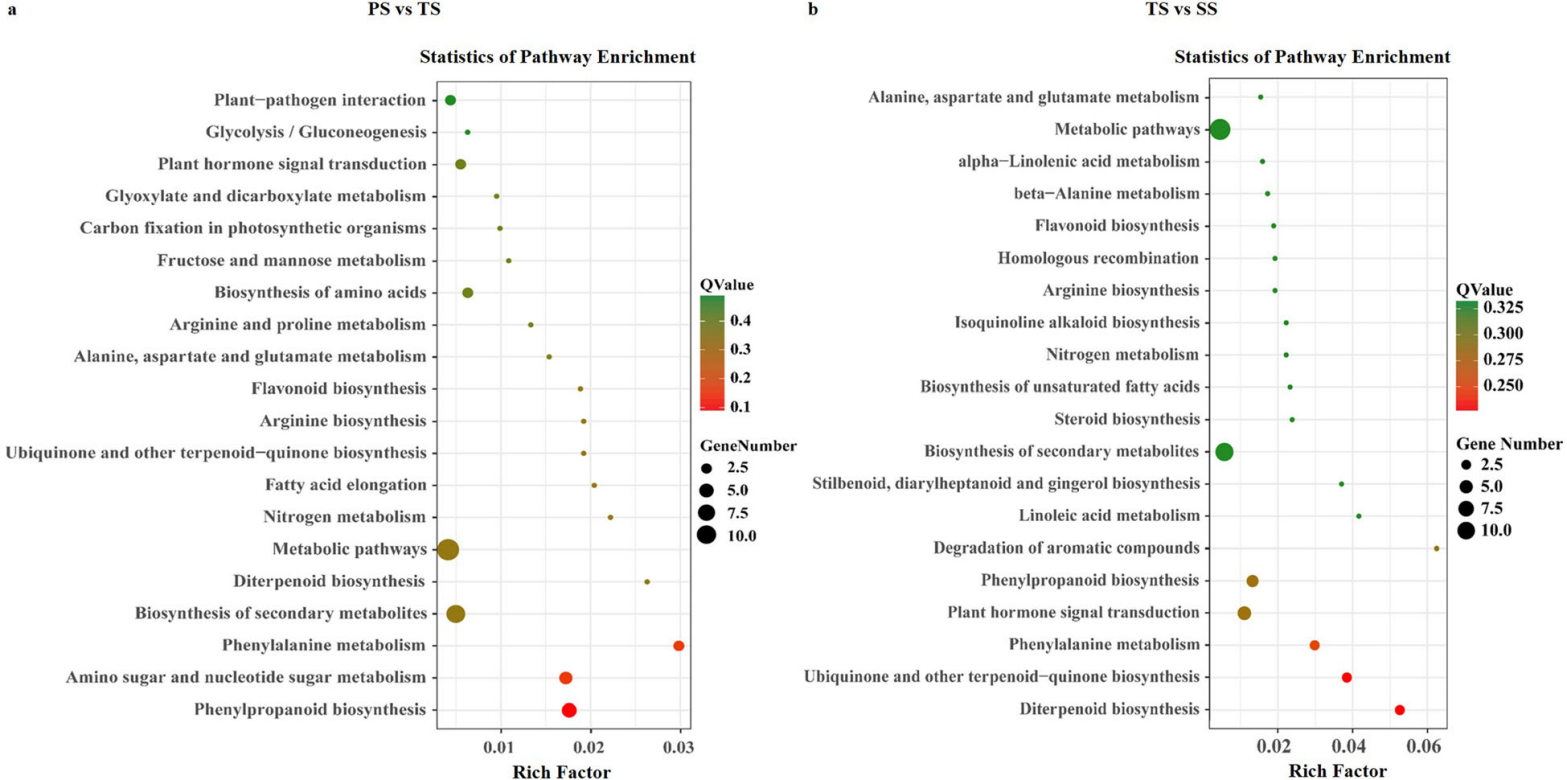
KEGG pathway enrichment of shared genes between differentially expressed genes (DEGs) and differentially methylated genes (DMGs) from primary stems (PS) versus transitional stems (TS) and TS versus secondary stems (SS). **a** KEGG pathway enrichment in PS vs TS. **b** KEGG pathway enrichment in TS vs SS. The size of each circle represents gene numbers, and the colors represent the q-values

Functional enrichment analysis on the 123 common genes revealed the enrichment of phenylpropanoid biosynthesis, phenylalanine metabolism, and the biosynthesis of secondary metabolites (Fig. 8a) from PS vs TS. KEGG pathway enrichment analyses on the rest of the DMGs from PS vs TS revealed the enrichment of pathways including pyrimidine metabolism, isoflavonoid biosynthesis, caffeine metabolism, and homologous recombination (Additional file 12A). Enrichment analyses with the DEGs from PS vs TS revealed pathways for phenylpropanoid biosynthesis, phenylalanine metabolism, and the biosynthesis of secondary metabolites (Additional file 12B), suggesting that DNA methylation participates in the initial stage of secondary cell wall formation. The same analysis was applied to the 114 genes common to DMGs and DEGs from TS vs SS, and the results showed that diterpenoid biosynthesis, ubiquinone and other terpenoid-quinone biosynthesis, phenylalanine metabolism, plant hormone signal transduction, and phenylpropanoid biosynthesis pathways were among those enriched (Fig. 8b). It was surprising that the enriched pathways in the rest of the DMGs from TS vs SS only consisted of brassinosteroid biosynthesis and caffeine metabolism pathways (Additional file 12C); the remaining DEGs from TS vs SS revealed multiple metabolic pathways, including biosynthesis of secondary metabolites, phenylpropanoid biosynthesis, and flavonoid biosynthesis (Additional file 12D), again indicating that DNA methylaton contributes to secondary wood formation and metabolites during the TS to SS transition.

Since transcription factors (TFs) lie at the center of gene regulation, we identified TFs with pronounced alternations in methylation levels. We found 32 TFs in the DMGs from PS vs TS and 39 TFs in the DMGs from TS vs SS which shared 11 TFs with the 229 DEGs from PS vs TS and 6 TFs with the 245 DEGs from TS vs SS (Additional files 13 and 14). For example, we found the TFs *NAC056*, *MYB52*, *NTL9*, *WRKY27*, and *MYB106* from PS vs TS (Additional file 13) and the TFs *bZIP21*, *MYB52*, *ANAC047*, and *HRD* from TS vs SS (Additional file 14).

## Discussion

Whole-genome bisulfite sequencing (WGBS) is a powerful technology for studying genome-wide DNA methylation patterns at single-nucleotide resolution. To date, an increasing number of plant species have been studied using WGBS, ranging from model species like *A. thaliana* [23, 24, 34] to some agriculturally important crops like rice [38, 39], maize [40], soybean [41], tomato [42], spruce [43], oil palm [44], and *P. trichocarpa* [45]. However, there is still a lack of understanding of the roles of methylation in secondary growth and development of tree species. Here, we decoded the single-base poplar stem methylome by WGBS and obtained an overall average of ~ 48.78%, ~ 30.39%, and ~ 5.72% methylation levels in CG, CHG, and CHH contexts (Additional file 1), respectively. Although methylation levels in poplar stems fell into the ranges described for other angiosperms (CG: ~ 30.5% to ~ 92.5%, CHG: ~ 9.3 to ~ 81.2%, and CHH: ~ 1.1% to ~ 18.8%) [46], they were on the lower end in each methylation context. The relatively low methylation levels in poplar stems maybe due, in part, to the fact that the *P. trichocarpa* genome (~ 422.9 Mb) is small compared to that of other plant species; previous studies have demonstrated that DNA methylation level is positively correlated with genome size [43, 46]. For example, apple, which has a moderately sized genome (~ 742 Mb), had ~ 53.6%, ~ 37.7%, and ~ 8.5% methylation levels in CG, CHG, and CHH contexts, respectively [47]. In *A. thaliana*, CG sites show a bimodal distribution and tend to be either unmethylated or highly methylated, whereas CHG and CHH sites are rarely methylated at high levels [23, 24]. This phenomenon implies that distinct mechanisms are responsible for maintaining different methylation types. As reported, CG methylation is copied faithfully during DNA replication, whereas CHG and CHH methylation are perpetually targeted by histone methylation and/or noncoding RNAs [13]. Interestingly, in *P. trichocarpa*, we found bimodal distribution patterns (unmethylated or methylated) for both CG and CHG methylation (Fig. 3a and b), suggesting that CHG methylation is maintained more robustly and copied more faithfully in poplar than in *A. thaliana*. Additionally, our results showed that methylated sites were concentrated on non-CG sites in the genome of *P. trichocarpa*, particularly on CHH sites, which is similar to observations in other trees [36, 48]. We also found that methylation levels of poplar stems were negatively correlated with gene numbers (Fig. 4), which was consistent with previous studies [49]. Furthermore, CG, CHG, and CHH methylation levels were positively correlated with TE density (Fig. 4) in the similar ways as what were demonstrated as in apple [47]. Some types of TEs, including LTR Copia and LTR Gypsy, are primarily distributed in pericentromeric regions of chromosomes [50]; we found that methylation of poplar stems also peaked in the centromere and pericentromeric regions as demonstrated in Fig. 3d and Additional files 2, 3, and 4. This finding has also been reported in other plant species [46, 47], indicating that hypermethylation may contribute to the maintenance of chromosomal stability and segregation.

Tissue differentiation coupled with distinct methylation levels is a universal phenomenon in all multicellular organisms. For example, methylation levels differ among seven distinct tissue types in *P. trichocarpa* [30], including vegetative bud, male inflorescence, female catkins, leaves, roots, xylem, and phloem, and in leaves [51], buds [52], and xylem [53] in natural populations of Chinese white poplar. Such a differentiation in methylation is essential for plant growth and development [54]. In this study, we found that the percentage of minimally methylated (0–10%) CG, CHG, and CHH sites decreased in TS and SS than PS (Fig. 3a, b and c). Therefore, mCG, mCHG, and mCHH sites could be established more stably in TS and SS. We also found that CG and CHH methylation levels showed significant differences among PS, TS, and SS. For CHG methylation, although PS showed more noticeable differences when compared with either TS or SS, there were no significant differences between TS and SS (Additional file 6). The methylation levels in CG contexts were highest in TS, and the methylation levels in CHH contexts were highest in SS (Fig. 5). These results suggest that, to some degree, genome methylation is in accord with wood formation in poplar stems and undergoes an obvious alteration at the initiation of secondary growth. It is well known that demethylases plays an important role in DNA methylation. In TS and SS, the reduced expression of demethylases (*PtrDME-A*, *PtrDME-B*, *PtrDEMETER-LIKE 2-A*, and *PtrDEMETER-LIKE 2-B*) might contribute to the increased methylation level (Fig. 2). Moreover, the enzymatic activities of two proteins, DRM1 and DRM2, are responsible for asymmetric CHH methylation [55]. *PtrDRM1/2-B* and *PtrDRM1/2-C* had significant higher expression levels in TS and SS (Fig. 2), respectively, than other developmental stages. The increased CHH methylation level may have something to do with the increased expression of *PtrDRM1/2* in TS and SS.

It has been reported that DNA methylation can repress gene expression [56]. However, the relationship between DNA methylation and gene transcription is more nuanced than initially realized. For example, rice promoter methylation repressed gene expression only in some heavily methylated gene loci; on the contrary, gene body methylation was positively, rather than negatively, correlated with gene expression [35]. In this study, we found that the relationship between DNA methylation and gene expression was complicated by genic regions, methylation contexts, and developmental states. Previous results showed that methylation tends to suppress gene transcription, and non-methylated genes had higher expression levels [31]. However, in our study, we found that the expressed genes in PS, TS, and SS had higher CG but lower CHG and CHH methylation levels in gene bodies than non-expressed genes (Fig. 6). In addition, Methylation in gene bodies corresponded more strongly with gene expression levels than methylation in either promoter or 2 kb downstream regions. In general, we found that, compared to expressed genes, non-expressed genes in PS, TS, and SS had the lowest CHH methylation in promoter regions, the highest CHG and CHH methylation levels in gene bodies, and the highest CG and CHG methylation levels in 2 kb downstream regions (Fig. 6). DNA methylation in promoters has been shown to affect gene expression levels, and the influence of promoter methylation on gene expression increases as the methylation level escalates [57–59]. In this study, we found that, regardless of developmental stage (PS, TS, and SS), CHH methylation levels in promoter regions positively corresponded to gene expression levels (Fig. 6c) and thus may be used as an epigenetic mark for gene expression in poplar stems. However, CHH methylation levels in the promoter regions of active genes were not consistently higher than those of other gene groups, particularly genes with moderate expression levels. Therefore, caution needs to be taken when CHH methylation is used as an epigenetic mark of gene expression.

We identified only 123 common genes in 653 DMGs and 4978 DEGs from PS vs TS, and 114 common genes in 858 DMGs and 4780 DEGs from TS vs SS (Fig. 7d and e), which suggests that developmental processes are coupled with 5mC to some degree. The counterparts of some common genes in *A. thaliana* are involved in cell growth (*CCR4* [60]*, MAP 70–1* [61]*,* and *UGT85A* [62]), secondary cell wall biosynthesis (*NAC056* [63]*, PAL1* [64], *FLA11* [65], *C4H* [66], and *MYB52* [67]), and hormone and signal transduction (*WRKY27* [68]*, UGT84B1* [69], *anac047* [70], *PIP1* [71]*,* and *CYP716A1* [72]) (Additional files 15 and 16), indicating that methylation can affect some individual pathway or regulatory genes involved in wood formation. For example, expression of *PtrPAL2* (Potri.008G038200) increased dramatically with the change of DNA methylation sites in PS vs TS (Additional file 15). As we also know, the deamination of phenylalanine by *PAL* is the first step in monolignol biosynthesis [64]. Some *PAL* genes are specifically expressed in differentiating xylem, while others are expressed in many other tissues. Homologs of *PtrPAL2* in *Populus fremontii × angustifolia* [73] and in *Populus* ESTs [74, 75] also suggests that it is xylem specific. *PtrC4H1* (Potri.013G157900) was also observed with the similar changes in TS vs SS (Additional file 16). *C4H* converts cinnamic acid into 4-hydroxycinnamic acid, a precursor for many phenylpropanoids including flavonoids, phytoalexins, and monolignols [76]. *PtrC4H1* transcripts are abundant in differentiating xylem, suggesting that it is important in monolignol biosynthesis [66]. In addition, our functional enrichment analysis showed that DNA methylation participated in the initial stage of secondary cell wall formation, but thereafter DNA methylation mainly influenced secondary metabolites. This was further verified by analyzing methylated TFs. Of the 32 and 39 methylated TFs identified from PS vs TS and TS vs SS, respectively, 11 are among the 229 DEGs from PS vs TS and 6 are among 245 DEGs from TS vs SS (Additional files 13 and 14). However, among these 16 methylated TFs, there were three *PtrMYB52* genes (Potri.008G089700, Potri.012G039400, and Potri.015G033600) whose counterparts in *A. thaliana* have been shown to regulate secondary cell wall formation [67]. This supports the idea that DNA methylation may affect wood formation by modulating transcription networks during the transition from primary growth to wood formation. According to an earlier study, differences in the expression of specific genes with unique methylation patterns, rather than relative methylation levels between the two tissue types, plays a critical role in wood biosynthesis [31]. The location of cytosine methylation within *MYB* and *NAC* genes might differentially affect the abundances of their transcripts, which is applicable to *PtrMYB52* gene too. Through integrated analysis of genomic, DNA methylomic, and transcriptomic differences between cultivated and wild rice, primary DNA sequence divergence has been shown to be the major determinant of methylational differences at the whole genome level, but DNA methylational differences alone can only account for limited gene expression variation between cultivated and wild rice [35]. Although we failed to detect large scale methylation of wood formation genes during the transition from primary to secondary growth, we still observed the role of methylation in wood formation. It is possible that methylation may also affect wood formation indirectly by modulating hormone and signaling transduction; as shown (Fig. 8), the common genes between DMGs and DEGs from a comparison of TS vs SS were enriched in hormone signal transduction pathways. There is evidence that DNA methylation can affect the signaling processes of various hormones, including salicylic acid (SA) under biotic stress [77] and auxin, abscisic acid (ABA), SA and ethylene under abiotic stress [47]. Changes in methylation were observed particularly in the bodies of expressed genes and to a lesser extent in transposable elements. Together, DEGs and DMRs were significantly enriched in genes related to phytohormone metabolism or signaling pathways [19], suggesting that an indirect influence of methylation on wood formation may exist.

## Conclusion

Our study provided DNA methylomes from multiple poplar stem tissues varying from predominantly primary to secondary growth, and then characterized the correction between DNA methylation with gene expression during *P. trichocarpa* stem development. Our results indicate that DNA methylation only marginally affects pathway genes and regulators involved in wood formation, suggesting that further studies of wood formation should lean towards studying the indirect effects of methylation. The information and data provided here will be instrumental for understanding the roles of methylation in wood formation in tree species.

## Methods

### Plant materials

A few plantlets of *P. trichocarpa* clone Nisqually-1, whose genome was sequenced [78] early, were obtained from the Shanghai Institute for Biological Sciences, Chinese Academy of Sciences, and vegetatively propagated in our lab using tissue culture [79]. The plantlets were planted in humus soil and grown under 16 h/8 h day/night photoperiod at 25 °C in the greenhouse at Northeast Forestry University for 90 days. Then, 63 trees were used as experimental materials for anatomical and histological analysis, transcriptomic profiling, and DNA methylation sequencing. The lengths of all internodes (IN) from the apical buds to the bases of the main stems of all trees were measured. As illustrated in Fig. 1a, the IN2, IN4, and IN8 internodes, representing the PS, TS, and SS stages from primary growth to secondary growth, respectively, were used for anatomical and histological analysis. Other internodes were immediately frozen in liquid nitrogen and stored at − 80 °C for qRT-PCR, RNA-seq, and DNA methylation sequencing. Three independent biological replicates were used for each of the above experiments.

### Anatomical and histological analysis of poplar stems

Approximately 3-mm-long segments of each internode sample from PS, TS, and SS were fixed in FAA buffer (50% ethanol, 5% acetic acid and 3.7% formaldehyde) and then embedded in paraffin. Transverse sections (8-μm thick) were cut from the embedded tissues with a sliding microtome (HM340E, Microm, Germany). Microscopic sections were stained with 0.025% (m/v) toluidine blue-O for 30 s, washed with dH_2_O, mounted on glass slides, and then examined using bright field microscopy (BX43, Olympus, Japan). The lignin present in the secondary walls in microsections was detected by histological straining with 2.5% phloroglucinol in 12 N HCl for 3 min and then observed using a microscope (Stereo Lumar.V12, Zeiss, Germany). Cellulose was stained with 0.25 μg/mL calcofluor white for 2 min and then visualized and photographed with a UV fluorescence microscope.

### DNA methylation analysis using qRT-PCR

Previous studies have shown that genes such as *MET1* [80], *DRM1*, *DRM2* [55], *CMT3* [81, 82], *DDM1* [83], *DME* [84], *DEMETER-LIKE2* [85], and *ROS1* [86] play crucial roles in maintaining various genomic methylation states. To determine whether the methylation states correspond to different developmental stages in poplar stems, the orthologs of the above genes in *P. trichocarpa* were retrieved from the Phytozome database [87] using a Basic Local Alignment Search Tool (BLAST) program named BLASTP. *DNA methyltransferase (PtrMET1A/B), DOMAINS REARRANGED METHYLTRANSFERASE 1 (PtrDRM1/2-A-C), CHROMOMETHYLASE 3 (PtrCMT3-A-C),* and *DECREASE IN DNA METHYLATION 1 (PtrDDM1-A/B)* are each involved in DNA methylation [88], and *DEMETER (DME) DNA demethylase* (*PtrDME-A/B)*, *PtrDEMETER-LIKE 2-A/B*, and *REPRESSOR OF SILENCING 1* (*PtrROS1)* are involved in DNA demethylation. The expression levels of these genes in PS, TS, and SS were analyzed using qRT-PCR, as in our previous study [89]. Transcript of *PtrActin* was used as an endogenous control to normalize expression in different samples. Primers used for these studies are listed in Additional file 17. The expression level of each gene relative to the reference gene was calculated using the delta-delta CT method. Each measurement was carried out with three biological replicates, and each biological replicate has three technical replicates. Each error bar represents a standard error (SE) of the mean fold changes of the three biological replicates.

### WGBS library construction and high-throughput sequencing

Plant samples were ground to powder in liquid nitrogen and genomic DNA was isolated using the DNeasy Plant Mini Kit (Qiagen China, Shanghai). The quantity and quality of extracted DNA were determined with a NanoDrop ND-1000 (Thermo, USA). Then, genomic DNA was fragmented to 100-300 bp by sonication (Covaris, Massachusetts, USA) and purified with a Mini Elute PCR Purification Kit (Qiagen, MD, USA). The fragmented DNAs were end-repaired and a single “A” nucleotide was added to the 3′ end of the blunt fragments. Then the genomic fragments were ligated to methylated sequencing adapters. Adaptor-added DNA was subjected to bisulfite conversion using the EZ DNA Methylation Gold Kit (Zymo Research), and the bisulfite-treated DNA was PCR amplified for 16 cycles. The resultant DNA was subjected to paired-end sequencing on an Illumina HiSeq 2500 sequencer; reads of 150 nucleotides were generated.

### Mapping and processing of BS-Seq reads

Raw read sequences generated by the Illumina pipeline in FastQ format were first subjected to quality control [90]. To get high quality clean reads, raw reads were filtered according to the following rules: 1) remove reads containing more than 10% unknown nucleotides (N); 2) remove low quality reads containing more than 40% low quality (Q-value ≤20) bases. Clean reads were then mapped to the *P. trichocarpa* reference genome using BSMAP software (version: 2.90) with default parameter [91]. Briefly, both clean reads and the reference genome were first transformed into bisulfite-converted versions (C-to-T and G-to-A converted). Then, the converted reads were aligned to the similar versions of the converted genome in a directional manner. To eliminate the bias produced by the alignment of duplicates generated by PCR, a de-duplication step was used to remove reads mapping to the same position of the reference genome. Finally, sequence reads that produced a unique best alignment from the two alignment processes (original top and bottom strand) were compared to the normal genomic sequence, and the methylation state of all cytosine positions in the read were inferred [92]. The bisulfite conversion rate of lambda DNA was calculated and used as a measure of the false discovery rate in the identification of the methylation site according to the binomial probability distribution. Each replicate (sample) was mapped to genome separately. After read mapping, we merged multiple replicates by running a 200 bp sliding window in a step of 100 bp. The methylation rates of the same windows, which have the same chromosomal position, across different replicates were merged to get the unified methylated cytosine percentage. The methylation levels of each of the three methylation types, CG, CHG, and CHH, were calculated in the whole genome, in each chromosome, and in different genomic regions. For all genic regions, methylation profiling of gene bodies and 2 kb flanking regions (both sides of the genes) were plotted based on the average methylation levels of different sliding windows.

### Identification of differentially methylated regions

Differential DNA methylation between the two samples at each locus was determined using Pearson’s chi-square test (χ2) in methyl Kit (version: 1.7.10) [93]. The minimum read coverage for a base to call a methylation status was set to 4. DMRs for each sequence context (CG, CHG, and CHH) conform to the following criteria: 1) For CG and CHG, numbers of GC or CHG in each window ≥5, absolute value of the difference in methylation ratio ≥ 0.25, and q ≤ 0.05; 2) For CHH, numbers in a window ≥15, absolute value of the difference in methylation ratio ≥ 0.15, and q ≤ 0.05; 3) For all C, numbers in a window ≥20, absolute value of the difference in methylation ratio ≥ 0.2, and q ≤ 0.05.

### Transcriptome sequencing and gene expression analysis

The same plant materials used for methylome analysis were also used for RNA isolation. The materials were first ground to powder in liquid nitrogen and total RNA was isolated using an RNA isolation kit (Auto Lab Biotechnology, Beijing, China). Using the RNase-Free DNase Set (Qiagen), we performed on-column DNase digestions three times during the RNA purification. The quantity and quality of extracted RNA were determined with a NanoDrop ND-1000 (Thermo, USA).

Whole transcriptome libraries were constructed using the NEB Next Ultra Directional RNA Library Prep Kit for Illumina (NEB, Ipswich, MA, USA) according to the manufacturer’s instructions; the resulting libraries were assessed for size, quantitation, integrity and purity using a Bioanalyzer 2100 system and qPCR (Kapa Biosystems, Woburn, MA, USA). The libraries with good quality were subsequently sequenced on a HiSeq 2500 instrument that was set to produce 125 bp paired-end reads of 125 nucleotide long. Raw sequences were cleaned as follows: 1) remove reads containing adapters; 2) remove reads containing more than 10% unknown nucleotides (N); 3) remove low quality reads containing more than 50% low quality (Q-value≤20) bases. After that, the clean reads from all the samples were mapped to the *P. trichocarpa* genome using Bowtie2 [94] and TopHat2 [95] software with default parameters [96]. The expression levels of the protein-coding genes were calculated and normalized using fragments per kilobase of gene per million mapped fragments (FPKM) by Cufflinks (version 2.2.1) [97].

Genes were divided into non-expressed genes (FPKM ≤1) and expressed genes. The latter were further divided into three groups based on expression levels, including minimally-expressed genes (Low, 1 < FPKM ≤10), moderately expressed genes (Moderate, 10 < FPKM ≤100), and highly expressed genes (High, FPKM ≥100).

### Relation between DNA methylation and gene expression

DMRs were annotated using the gene annotation file P*.*trichocarpa_444_v3.1.gene.gff3.gz from the *P. trichocarpa* reference genome provided by Phytozome [78, 87]. The positions and types of TEs were obtained from Ptrichocarpa_533_v4.1.repeatmasked_assembly_v4.0.gff3.gz downloaded from the Joint Genome Institute website (https://genome.jgi.doe.gov/portal/pages/dynamic OrganismDownload.jsf?organism=Phytozome) [37]. Annotations were done by comparing the chromosome position information of DMRs with the corresponding annotation information in the gene annotation file. When a DMR overlapped a gene (> 1 nt), including a gene body and the 2 kb flanking region on either side, the DMR was associated with this gene and was then designated a DMG. Spearman correlation analysis was performed to discern statistical relationship between DNA methylation and gene expression within gene bodies and their ±2 kb flanking regions. To explore the potential contribution of DNA methylation to the differentiation of gene expression, DMGs, DEGs, and common genes between DMGs and DEGs were subjected to KEGG pathway enrichment analysis (KEGG: http://www.genome.jp/kegg/). KOBAS software [98] was used to test the statistical enrichment of DEGs in various KEGG pathways.

### Data analysis

The data were analyzed using one-way ANOVA and Duncan’s multiple range test using SPSS 21 (Chicago, IL, USA). A statistically significant level was set to a *p* value < 0.05. The data are presented as mean ± standard error (SE) with each SE being calculated from three independents biological samples.

## Supplementary information

**Additional file 1. **Summary of the methylation ratio in different sequence contexts (CG, CHG, and CHH) in developmental stems of *Populus trichocarpa.*

**Additional file 2.** Distributions of 5-methylcytosine density on Chromosomes 2–19 in primary stems (PS).

**Additional file 3.** Distributions of 5-methylcytosine density on Chromosomes 1–19 on transitional stems (TS).

**Additional file 4.** Distributions of 5-methylcytosine density on Chromosomes 1–19 on secondary stems (SS).

**Additional file 5. **Relative proportions of mCs in three sequence contexts (CG, CHG and CHH) in *Populus trichocarpa* stems.

**Additional file 6. **Comparison of mCs in CG, CHG, and CHH sequence contexts in three developmental stages: primary stems (PS), transitional stems (TS) and secondary stems (SS). The y-axis indicates the percentage of mC (%). The different letters on the top of the error bars indicate statistically significant differences between means at *p* < 0.05.

**Additional file 7. **Relative percentages of 5-mCs in three sequence contexts (CG, CHG, and CHH) in various transposable elements (TEs) of *Populus trichocarpa* stems.

**Additional file 8.** Methylation levels in various transposable elements (TEs) and their 2 kb proximal regions in primary stems (PS), transitional stems (TS), and secondary stems (SS). The y-axis represents methylation levels.

**Additional file 9. **Comparison of methylation levels in different developmental stages: primary stems (PS), transitional stems (TS), and secondary stems (SS). The y-axis represents methylation levels. The different letters on the top of the error bars indicate statistically significant differences between means at *p* < 0.05.

**Additional file 10.** Relationship between gene methylation (5-methylcytosines) and gene expression. (A), (B), and (C) Expression profiles of methylated genes compared with unmethylated genes in CG, CHG, and CHH contexts in primary stems (PS), respectively. (D), (E), and (F) Expression profiles of methylated genes compared with unmethylated genes in CG, CHG, and CHH contexts in transitional stems (TS), respectively. (G), (H), and (I) Expression profiles of methylated genes compared with unmethylated genes in CG, CHG, and CHH contexts in secondary stems (SS), respectively. The y-axis indicates the methylation levels. None represents the unmethylated group. Low represents the bottom third of the methylation group. Moderate represents the middle third of the methylation group. High represents the top third of the methylation group.

**Additional file 11.** Correlation between 5-methylcytosine methylation in different genic regions and gene expression in primary stems (PS), transitional stems (TS), and secondary stems (SS) of poplar. (A), (B), and (C) represent CG, CHG, and CHH DNA methylation contexts, respectively. The regions of promoter, gene body, and 2 kb downstream regions were split on the x-axis to investigate the Spearman rank correlation (y-axis) between levels of methylation and expression. Rho > 0 means positive correlation, and rho < 0 means negative correlation.

**Additional file 12.** KEGG pathway enrichment of the rest of the differentially methylated genes (DMGs) and differentially expressed genes (DEGs). (A) and (B) represent KEGG pathway enrichment of the remaining DMGs and DEGs in primary stems (PS) vs transitional stems (TS), respectively. (C) and (D) represent KEGG pathway enrichment of the remaining DMGs and DEGs in TS vs secondary stems (SS), respectively. The size of the circle represents gene numbers, and the colors represents the q-value.

**Additional file 13.** The transcription factors (TFs) in primary stems (PS) vs transitional stems (TS).

**Additional file 14.** The transcription factors (TFs) in transitional stems (TS) vs secondary stems (SS).

**Additional file 15.** Shared genes in differentially expressed genes (DEGs) and differentially 5-methylcytosine methylated genes (DMGs) in primary stems (PS) vs transitional stems (TS).

**Additional file 16.** Shared genes in differentially expressed genes (DEGs) and differentially 5-methylcytosine methylated genes (DMGs) in transitional stems (TS) vs secondary stems (SS).

**Additional file 17.** Homologs of DNA methyltransferase and demethylase proteins in poplar.

## Data Availability

The datasets used and analysed in this study are available in Sequence Read Archive (SRA) at NCBI at National Center for Biotechnology Information. The accession numbers for methylation data and RNA-seq data are PRJNA628812 and PRJNA628501, respectively. The data sets can be accessed at SRA (https://www.ncbi.nlm.nih.gov/sra) with the accession number provided above or https://www.ncbi.nlm.nih.gov/bioproject/PRJNA628812 and https://www.ncbi.nlm.nih.gov/ bioproject/PRJNA628501.

## Abbreviations

5mC: 5-methylcytosine
LTR: Long terminal repeats
IN: Internode
PS: Primary stems
TS: Transitional stems
SS: Secondary stems
WGBS: Whole-genome bisulfite sequencing
TEs: Transposable elements
LINE: Long interspersed nuclear elements
SINE: Short interspersed nuclear elements
DMRs: Differentially methylated regions
DMGs: Differentially methylated genes
DEGs: Differentially expressed genes
TFs: Transcription factors
SA: Salicylic acid
ABA: Abscisic acid
BLAST: Basic Local Alignment Search Tool
