# Supplementary figures and images for: DNA methylation and its effects on gene expression during primary to secondary growth in poplar stems
Source: BMC Genomics. 2020 Jul 20;21:498. doi: 10.1186/s12864-020-06902-6 (PMC7372836; doi:10.1186/s12864-020-06902-6)

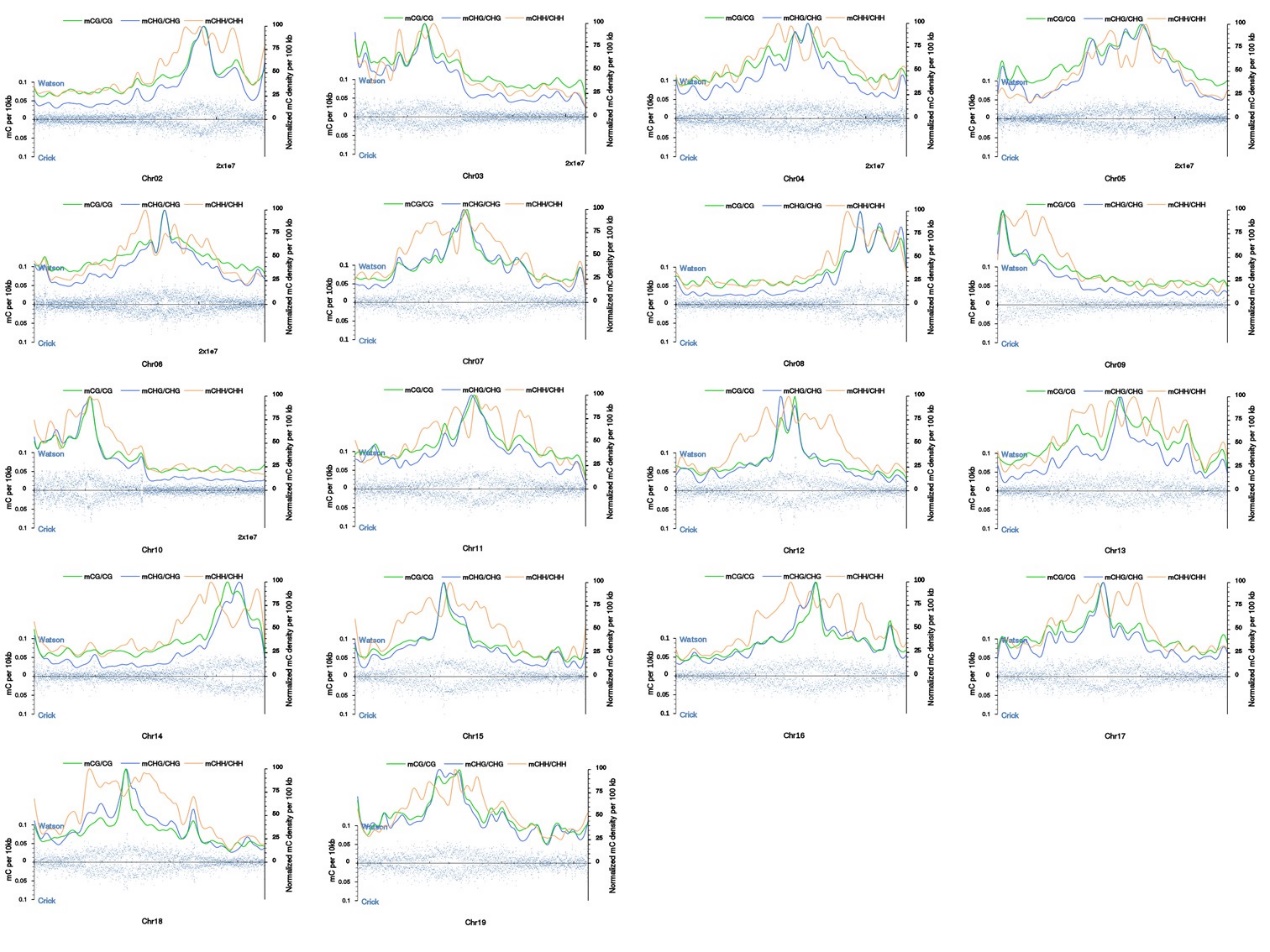


**Additional file 2 Distributions of 5-methylcytosine density on chromosomes 2-19 in primary stems (PS).**

Supplement: Supplementary file 2 — Additional file 2. Distributions of 5-methylcytosine density on Chromosomes 2–19 in primary stems (PS). [file 12864_2020_6902_MOESM2_ESM.docx]
