# Supplementary material for: DNA methylation and its effects on gene expression during primary to secondary growth in poplar stems
Source: BMC Genomics. 2020 Jul 20;21:498. doi: 10.1186/s12864-020-06902-6 (PMC7372836; doi:10.1186/s12864-020-06902-6)
Supplement: Supplementary file 4 — Additional file 4. Distributions of 5-methylcytosine density on Chromosomes 1–19 on secondary stems (SS). [file 12864_2020_6902_MOESM4_ESM.docx]

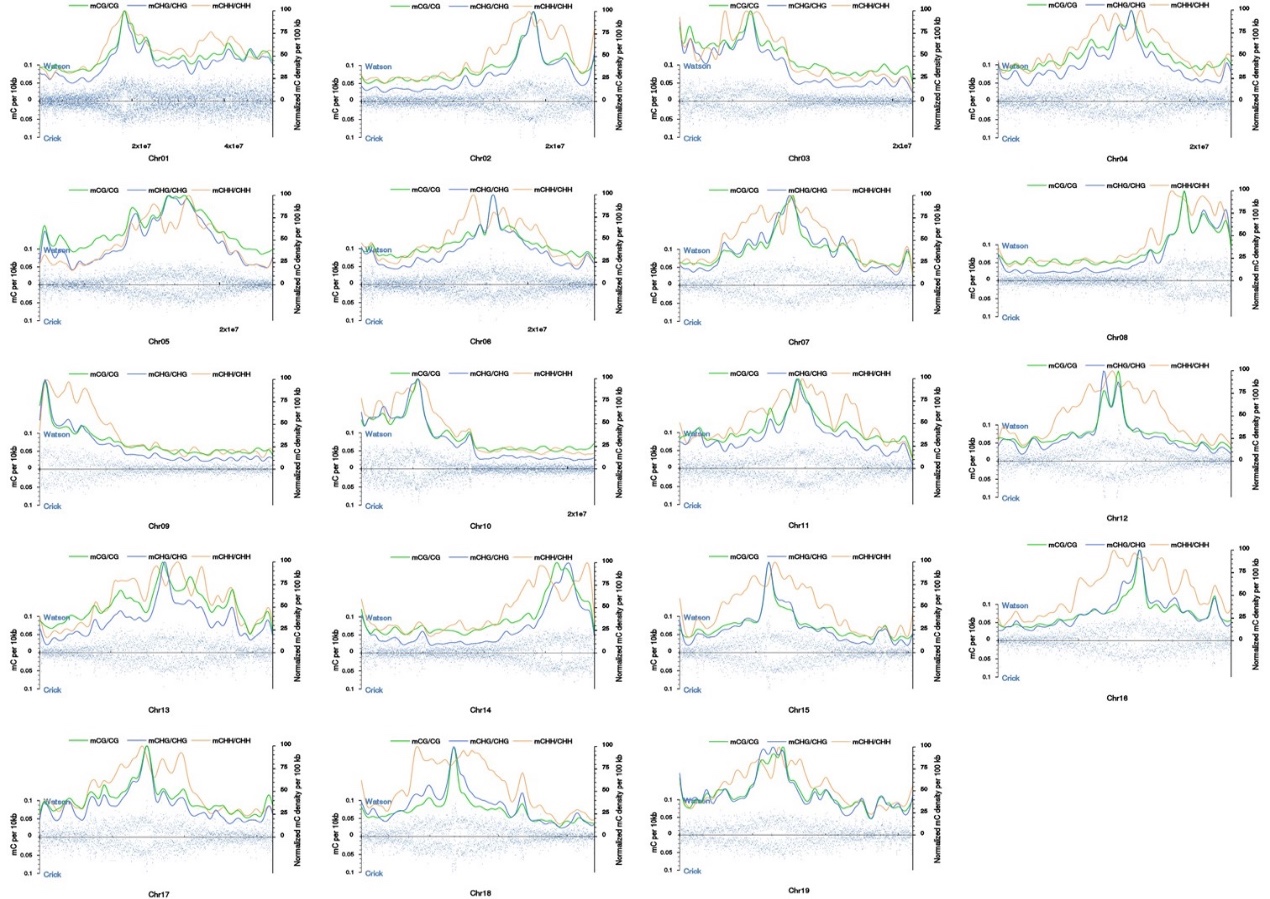


**Additional file 4 Distributions of 5-methylcytosine density on chromosomes 1-19 on secondary stems (SS).**
