# Supplementary material for: DNA methylation and its effects on gene expression during primary to secondary growth in poplar stems
Source: BMC Genomics. 2020 Jul 20;21:498. doi: 10.1186/s12864-020-06902-6 (PMC7372836; doi:10.1186/s12864-020-06902-6)
Supplement: Supplementary file 8 — Additional file 8. Methylation levels in various transposable elements (TEs) and their 2 kb proximal regions in primary stems (PS), transitional stems (TS), and secondary stems (SS). The y-axis represents methylation levels. [file 12864_2020_6902_MOESM8_ESM.docx]

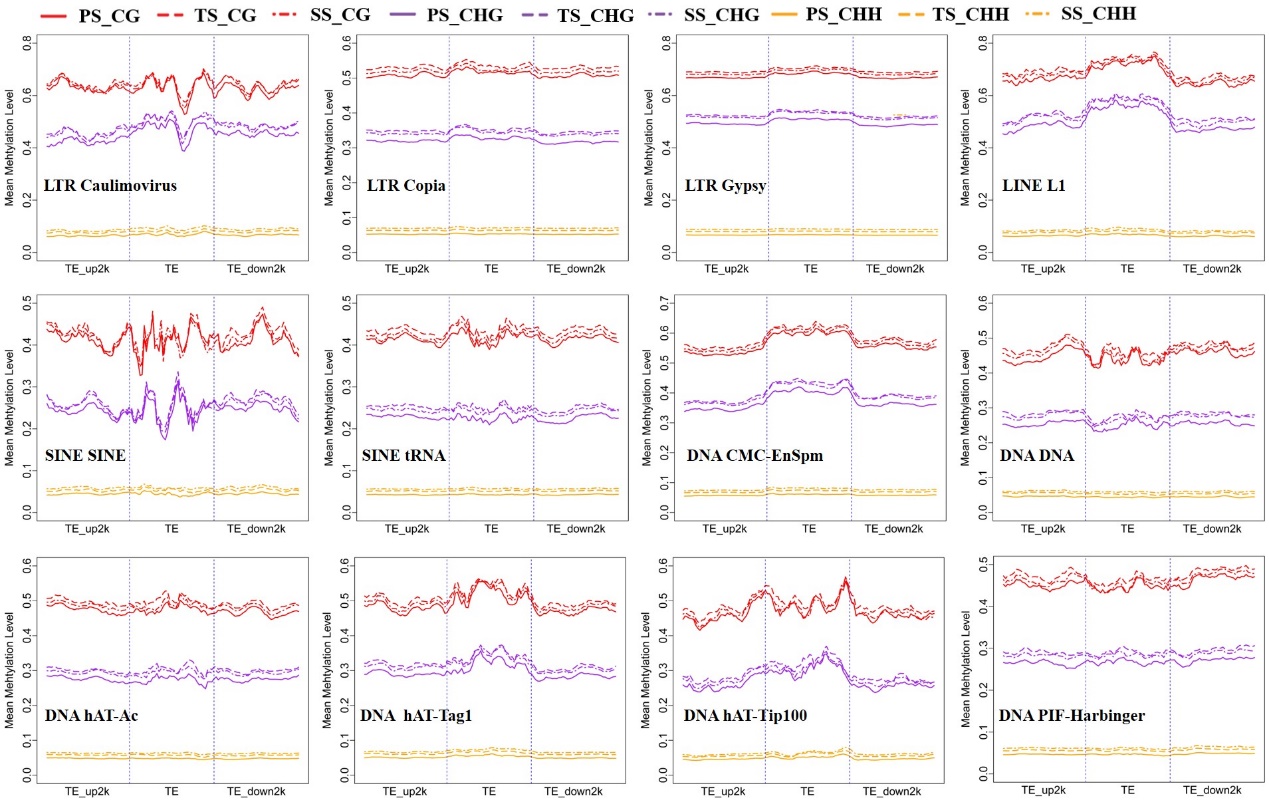


**Additional file 8 Methylation levels in various transposable elements (TEs) and their 2 kb proximal regions in primary stems (PS), transitional stems (TS), and secondary stems (SS).** The y-axis represents methylation levels.
