# Supplementary material for: DNA methylation and its effects on gene expression during primary to secondary growth in poplar stems
Source: BMC Genomics. 2020 Jul 20;21:498. doi: 10.1186/s12864-020-06902-6 (PMC7372836; doi:10.1186/s12864-020-06902-6)
Supplement: Supplementary file 9 — Additional file 9. Comparison of methylation levels in different developmental stages: primary stems (PS), transitional stems (TS), and secondary stems (SS). The y-axis represents methylation levels. The different letters on the top of the error bars indicate statistically significant differences between means at p < 0.05. [file 12864_2020_6902_MOESM9_ESM.docx]

**
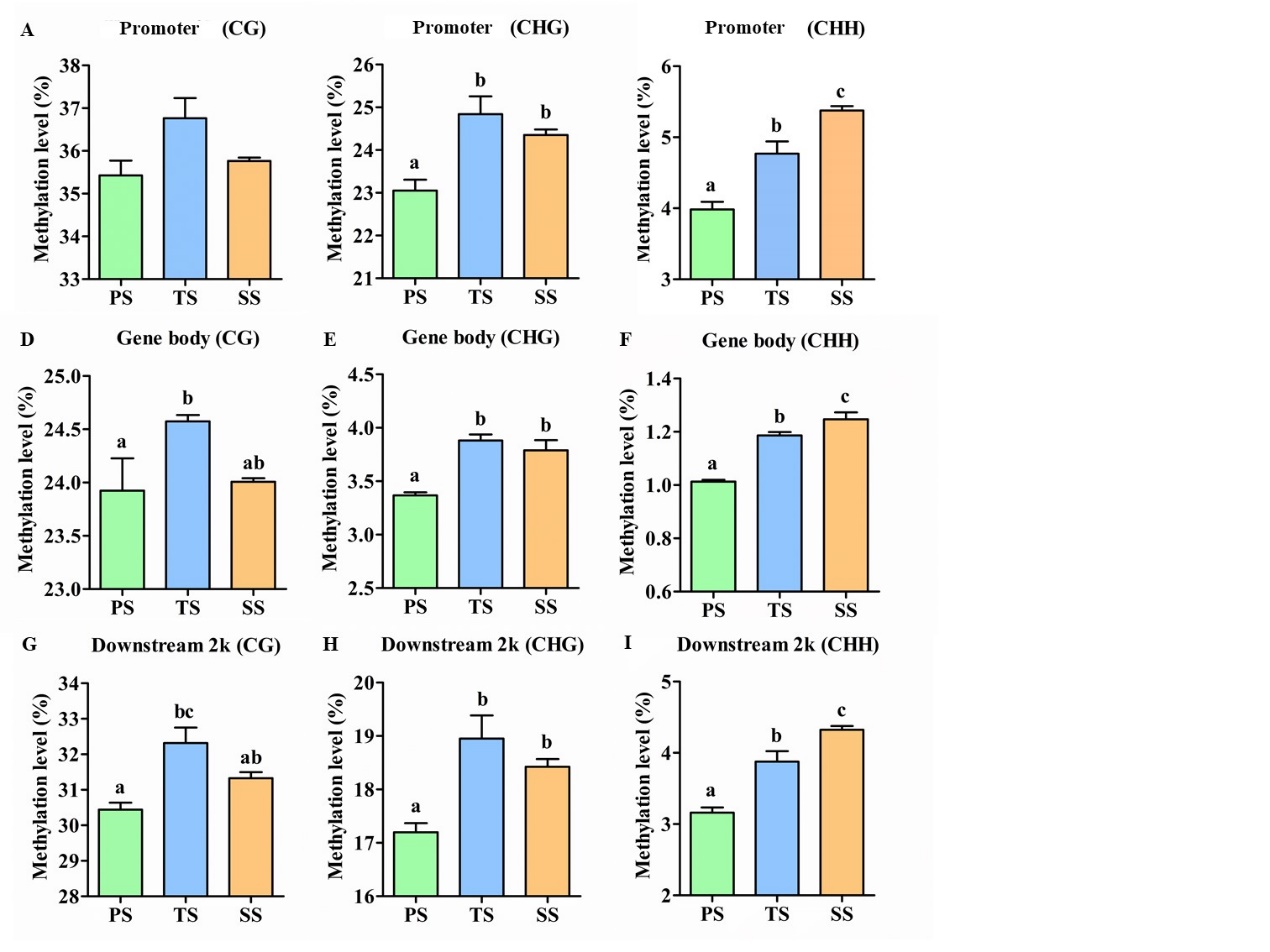
**

**Additional file 9 Comparison of methylation levels in different developmental stages: primary stems (PS), transitional stems (TS) and secondary stems (SS).** The y-axis represents methylation levels. The different letters on the top of the error bars indicate statistically significant differences between means at *p* < 0.05.
