# Supplementary material for: DNA methylation and its effects on gene expression during primary to secondary growth in poplar stems
Source: BMC Genomics. 2020 Jul 20;21:498. doi: 10.1186/s12864-020-06902-6 (PMC7372836; doi:10.1186/s12864-020-06902-6)
Supplement: Supplementary file 10 — Additional file 10. Relationship between gene methylation (5-methylcytosines) and gene expression. (A), (B), and (C) Expression profiles of methylated genes compared with unmethylated genes in CG, CHG, and CHH contexts in primary stems (PS), respectively. (D), (E), and (F) Expression profiles of methylated genes compared with unmethylated genes in CG, CHG, and CHH contexts in transitional stems (TS), respectively. (G), (H), and (I) Expression profiles of methylated genes compared with unmethylated genes in CG, CHG, and CHH contexts in secondary stems (SS), respectively. The y-axis indicates the methylation levels. None represents the unmethylated group. Low represents the bottom third of the methylation group. Moderate represents the middle third of the methylation group. High represents the top third of the methylation group. [file 12864_2020_6902_MOESM10_ESM.docx]

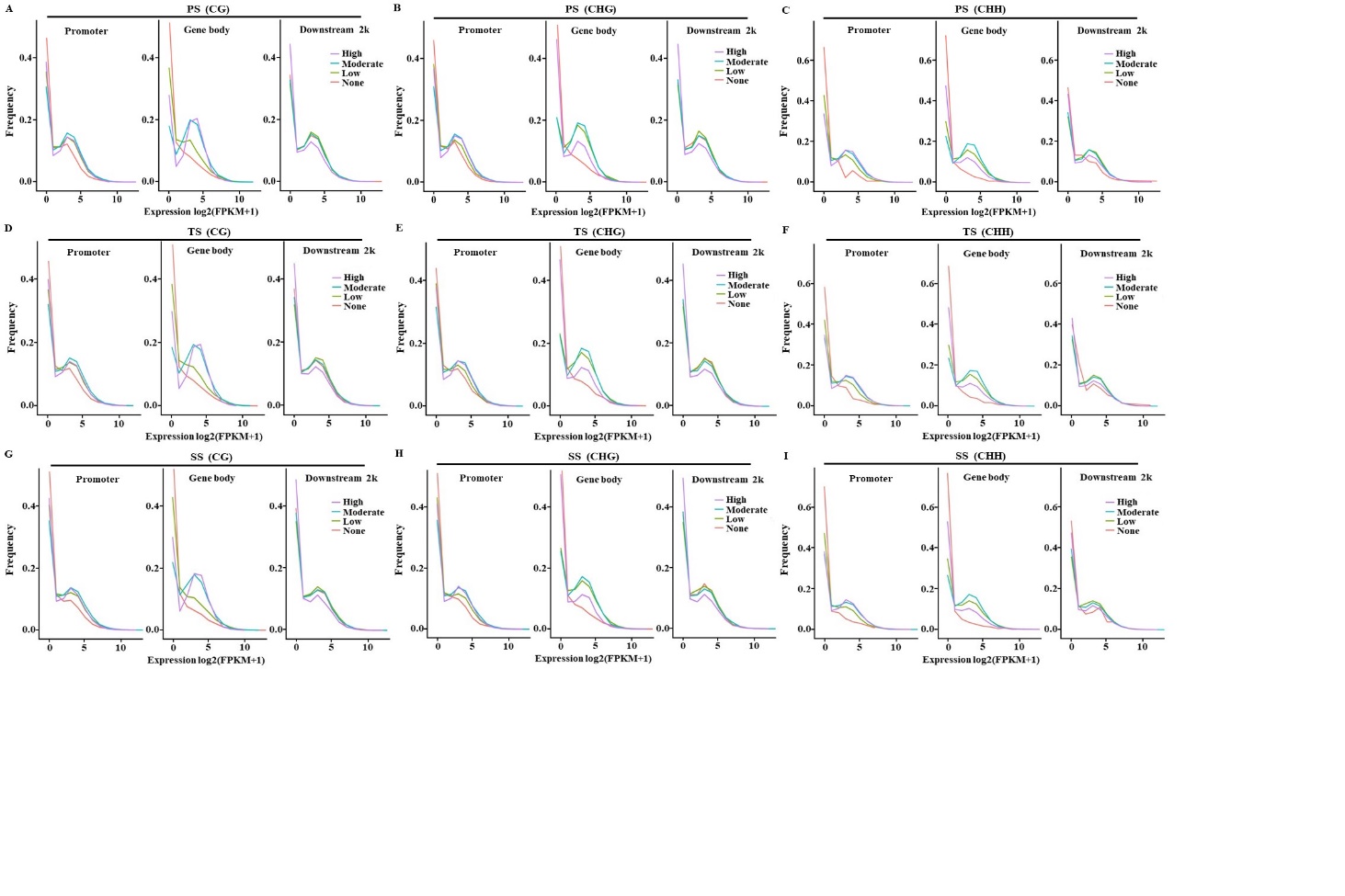


**Additional file 10 Relationship between gene methylation (5-methylcytosines) and gene expression. (A)**, **(B)**, and **(C)** Expression profiles of methylated genes compared with unmethylated genes in CG, CHG, and CHH contexts in primary stems (PS), respectively. **(D)**, **(E)**, and **(F)** Expression profiles of methylated genes compared with unmethylated genes in CG, CHG, and CHH contexts in transitional stems (TS), respectively. **(G)**, **(H)**, and **(I)** Expression profiles of methylated genes compared with unmethylated genes in CG, CHG, and CHH contexts in secondary stems (SS), respectively. The y-axis indicates the methylation levels. None represents the unmethylated group. Low represents the bottom third of the methylation group. Moderate represents the middle third of the methylation group. High represents the top third of the methylation group.
