# Supplementary material for: DNA methylation and its effects on gene expression during primary to secondary growth in poplar stems
Source: BMC Genomics. 2020 Jul 20;21:498. doi: 10.1186/s12864-020-06902-6 (PMC7372836; doi:10.1186/s12864-020-06902-6)
Supplement: Supplementary file 11 — Additional file 11. Correlation between 5-methylcytosine methylation in different genic regions and gene expression in primary stems (PS), transitional stems (TS), and secondary stems (SS) of poplar. (A), (B), and (C) represent CG, CHG, and CHH DNA methylation contexts, respectively. The regions of promoter, gene body, and 2 kb downstream regions were split on the x-axis to investigate the Spearman rank correlation (y-axis) between levels of methylation and expression. Rho > 0 means positive correlation, and rho < 0 means negative correlation. [file 12864_2020_6902_MOESM11_ESM.docx]

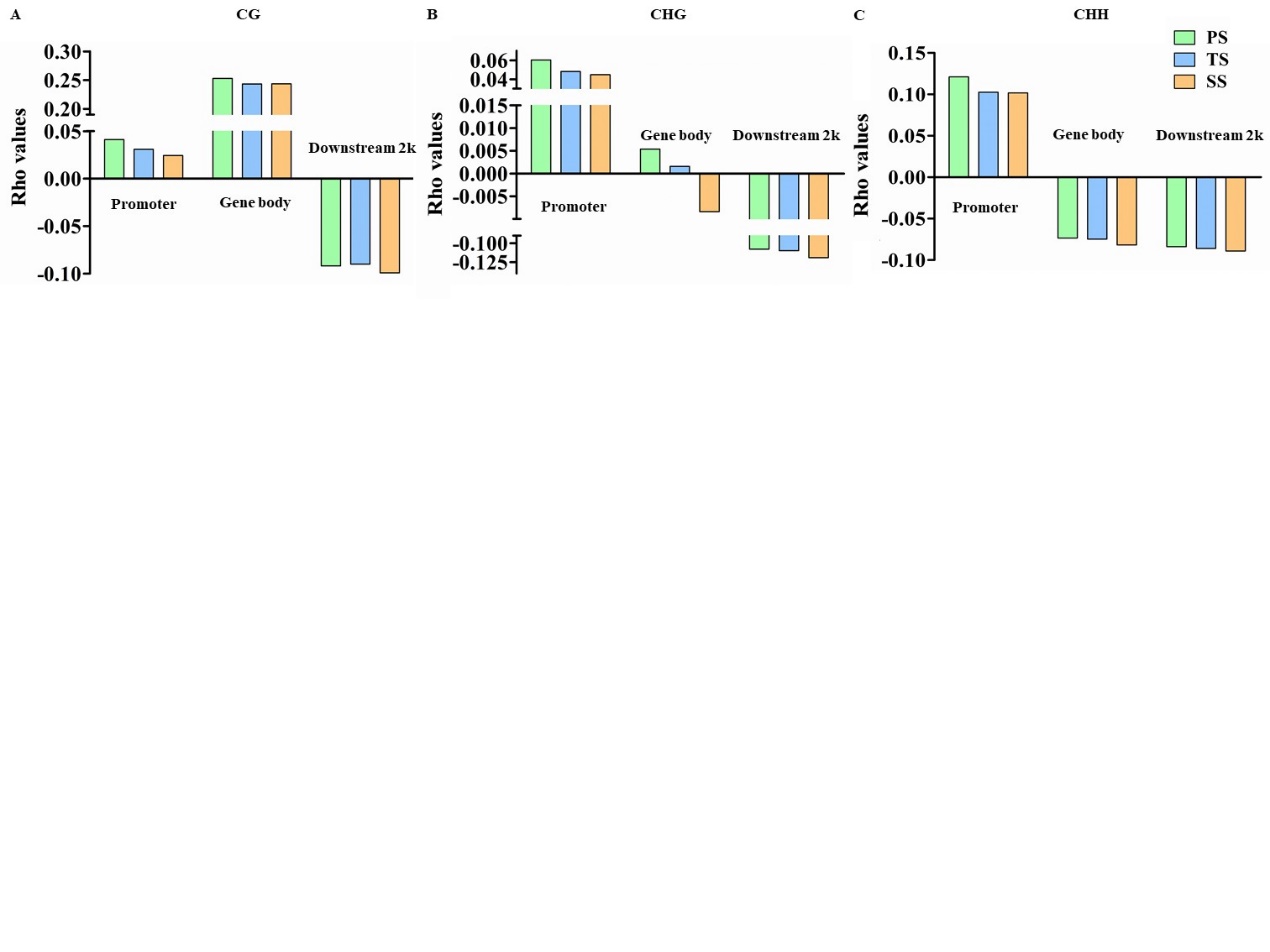


**Additional file 11 Correlation between 5-methylcytosines methylation in different genic regions and gene expression in primary stems (PS), transitional stems (TS), and secondary stems (SS) of poplar.** (A), (B), and (C) represent CG, CHG, and CHH DNA methylation contexts, respectively. The regions of promoter, gene body, and 2kb downstream regions were split on the x-axis to investigate the Spearman rank correlation (y-axis) between levels of methylation and expression. Rho > 0 means positive correlation, and rho < 0 means negative correlation.
