# Supplementary material for: DNA methylation and its effects on gene expression during primary to secondary growth in poplar stems
Source: BMC Genomics. 2020 Jul 20;21:498. doi: 10.1186/s12864-020-06902-6 (PMC7372836; doi:10.1186/s12864-020-06902-6)
Supplement: Supplementary file 12 — Additional file 12. KEGG pathway enrichment of the rest of the differentially methylated genes (DMGs) and differentially expressed genes (DEGs). (A) and (B) represent KEGG pathway enrichment of the remaining DMGs and DEGs in primary stems (PS) vs transitional stems (TS), respectively. (C) and (D) represent KEGG pathway enrichment of the remaining DMGs and DEGs in TS vs secondary stems (SS), respectively. The size of the circle represents gene numbers, and the colors represents the q-value. [file 12864_2020_6902_MOESM12_ESM.docx]

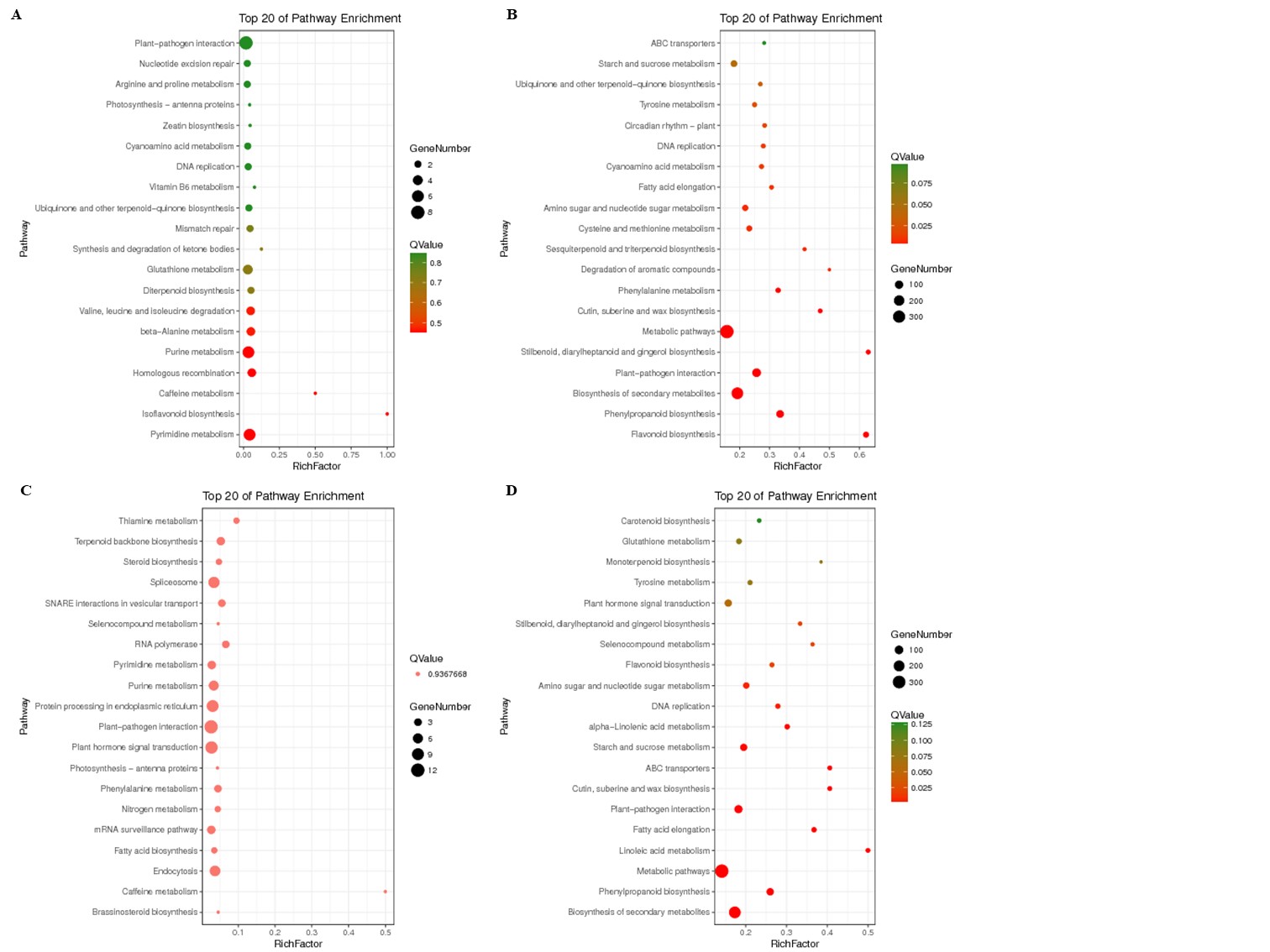


**Additional file 12 KEGG pathway enrichment of** **the rest of the differentially** **methylated genes (DMGs) and differentially expressed genes (DEGs).** (A) and (B) represent KEGG pathway enrichment of the remaining DMGs and DEGs in primary stems (PS) vs transitional stems (TS), respectively. (C) and (D) represent KEGG pathway enrichment of the remaining DMGs and DEGs in TS vs secondary stems (SS), respectively. The size of the circle represents gene numbers, and the colors represents the q-value.
